# Understanding the function of Pax5 in development of docetaxel-resistant neuroendocrine-like prostate cancers

**DOI:** 10.1038/s41419-024-06916-y

**Published:** 2024-08-25

**Authors:** Sreyashi Bhattacharya, Hannah L. Harris, Ridwan Islam, Sanika Bodas, Navatha Polavaram, Juhi Mishra, Dipanwita Das, Parthasarathy Seshacharyulu, Achyuth Kalluchi, Anirban Pal, Manish Kohli, Subodh M. Lele, Michael Muders, Surinder K. Batra, Paramita M. Ghosh, Kaustubh Datta, M. Jordan Rowley, Samikshan Dutta

**Affiliations:** 1 Department of Biochemistry and Molecular Biology, University of Nebraska Medical Center, Omaha, NE USA; 2 Department of Genetics, Cell Biology and Anatomy, University of Nebraska Medical Center, Omaha, NE USA; 3 Department of Biochemistry and Molecular Biology, Virginia Commonwealth University, Richmond, VA USA; 4 Data Scientist, Gallup, Omaha, NE USA; 5 School of Medicine, Division of Oncology, Huntsman Cancer Institute, Salt Lake City, UT USA; 6 MVZ Pathology Bethesda, Heerstrasse 219, Duisburg, Germany; 7 Department of Urological Surgery, University of California Davis, School of Medicine, Sacramento, CA USA

**Keywords:** Prostate cancer, Cancer therapeutic resistance

## Abstract

Resistance to the current Androgen Receptor Signaling Inhibitor (ARSI) therapies has led to higher incidences of therapy-induced neuroendocrine-like prostate cancer (t-NEPC). This highly aggressive subtype with predominant small-cell-like characteristics is resistant to taxane chemotherapies and has a dismal overall survival. t-NEPCs are mostly treated with platinum-based drugs with a combination of etoposide or taxane and have less selectivity and high systemic toxicity, which often limit their clinical potential. During t-NEPC transformation, adenocarcinomas lose their luminal features and adopt neuro-basal characteristics. Whether the adaptive neuronal characteristics of t-NEPC are responsible for such taxane resistance remains unknown. Pathway analysis from patient gene-expression databases indicates that t-NEPC upregulates various neuronal pathways associated with enhanced cellular networks. To identify transcription factor(s) (TF) that could be important for promoting the gene expression for neuronal characters in t-NEPC, we performed ATAC-Seq, acetylated-histone ChIP-seq, and RNA-seq in our NE-like cell line models and analyzed the promoters of transcriptionally active and significantly enriched neuroendocrine-like (NE-like) cancer-specific genes. Our results indicate that Pax5 could be an important transcription factor for neuronal gene expression and specific to t-NEPC. Pathway analysis revealed that Pax5 expression is involved in axonal guidance, neurotransmitter regulation, and neuronal adhesion, which are critical for strong cellular communications. Further results suggest that depletion of Pax5 disrupts neurite-mediated cellular communication in NE-like cells and reduces surface growth factor receptor activation, thereby, sensitizing them to docetaxel therapies. Moreover, t-NEPC-specific hydroxymethylation of Pax5 promoter CpG islands favors Pbx1 binding to induce Pax5 expression. Based on our study, we concluded that continuous exposure to ARSI therapies leads to epigenetic modifications and Pax5 activation in t-NEPC, which promotes the expression of genes necessary to adopt taxane-resistant NE-like cancer. Thus, targeting the Pax5 axis can be beneficial for reverting their taxane sensitivity.

## Introduction

Hormone sensitive recurrent or metastatic prostate cancer is preferentially treated with androgen deprivation therapies (ADT) alone or in combination with androgen receptor signaling axis inhibitors (ARSIs) such as abiraterone acetate or enzalutamide [1, 2]. However, over the time, most of these patients become resistant to ARSI therapies and progress to castration-resistant prostate cancer (CRPC) [1, 3]. CRPC is highly heterogeneous but still predominantly adenocarcinoma in nature, which are generally, treated with first-line taxane-based chemotherapies following ARSI resistance [4–6]. However, ~20% of these ARSI-resistant cases showed neuroendocrine-like transformation [therapy-induced neuroendocrine-like prostate cancer (t-NEPC) or neuroendocrine-like cancer (NE-like)], and often presented as small-cell-like neuroendocrine characteristics [7–10]. Similar to de novo NEPC, t-NEPCs are highly aggressive, readily metastasize to visceral organs and share common features including expression of neuroendocrine-specific genes [10–12]. However, t-NEPCs are resistant to taxane-based therapies and are preferentially treated with platinum-based drugs in combination with etoposides [13–15]. Compared to well-tolerated taxanes, these platinum-based therapies yield multiple systemic toxicities and fail to show much improvement on overall survival of t-NEPC patients [13, 14]. Currently, various clinical trials are ongoing to test the efficacy of targeted therapies; however, results are mostly inconclusive [16]. In this background, identification of key molecular regulator/s or pathways responsible for taxane resistance of t-NEPC will be important for future therapeutic optimization.

Studies have shown that functional aberrations of tumor suppressors RB1 and/or TP53 are important for NE-like trans-differentiation; however, why losses of RB1 and TP53 induce t-NEPC differentiation, remains poorly understood [17, 18]. While studying the differential characteristics between CRPC-adenocarcinoma and t-NEPCs, we found that t-NEPCs selectively upregulate genes related to the neuronal pathways [19]. Whether these neuronal phenotypes contribute to the taxane resistance of t-NEPC is unknown and demands clarification.

To replicate the trans-differentiation processes, we previously generated and characterized various t-NEPC models from established CRPC cell lines [19]. In this study, we have identified Pax5 as an important transcriptional regulator involved in various neuronal pathways. Pax5 expression is highly specific to t-NEPC, and depletion of Pax5 abrogates neuronal characteristics of t-NEPC. The present study highlights the importance of Pax5-mediated development of neuronal characteristics towards taxane resistance in t-NEPC. Furthermore, we discovered the importance of t-NEPC-specific epigenetic modification-based transcriptional events in Pax5 expression. Overall, the current work emphasizes the role of Pax5 transcriptional signature as a crucial element in neuronal gene expression linked to therapy-resistant prostate cancer.

## Materials and methods

### Reagents

Cell culture media- RPMI 1640 (Thermo Fisher Scientific, Gibco, NY, 11875093), DPBS, 0.25% (w/v) Trypsin, (100X), and Penicillin-Streptomycin (5000 U/ml) were purchased from ThermoFisher Scientific. Fetal bovine serum was purchased from GIBCO. For immunohistochemistry (IHC), biotin-conjugated goat anti-rabbit IgG (Invitrogen, 31820) was used as a secondary antibody followed by Reagent A: Avidin (Thermo Scientific, 1852280) and Reagent B: Biotinylated HRP (Thermo Scientific, 1852310). ImPACT DAB (Vector Laboratories, SK-4105) was used for visualizing the protein. For immunofluorescence and immunocytochemistry, secondary Alexa Fluor 594 goat anti-mouse (Thermo Fisher A11020), and Alexa Fluor 488 goat anti-rabbit (Thermo Fisher A11008) antibodies were used. Other reagents such as HEPES, KCl, DTT, NP-40, Glycerol, MgCl2, EDTA, PMSF, protease inhibitors such as aprotinin, and leupeptin were purchased from Sigma-Aldrich. Halt phosphatase inhibitor (1862495), Trizol and Powerup SYBR Green master mix were purchased from ThermoFisher Scientific. cDNA kit was purchased from Roche. All the primers were from IDT.

### Patient cohort for in-silico analysis

RNA-Seq data of the following patient cohorts were selected from public domains for analysis. Treatment-resistant mCRPC patient cohort with PCa patients having NE-like development (GSE 126078) [1], SU2C-PCF (Stand Up to Cancer/Prostate Cancer Foundation) International Prostate Cancer Dream Team consortium [20], a prospective clinical trial (identifier: NCT02432001) [17] and mCRPC patient cohort with CRPC-adeno and CRPC-neuro phenotype [10], GSE 137829 [21]. Gene expression profiles of GSE 126078, GSE 66187, GSE 137829, SU2C-PCF cohort (Stand Up to Cancer/Prostate Cancer Foundation) International Prostate Cancer Dream Team consortium were downloaded from Gene Expression Omnibus (GEO) (https://www.ncbi.nlm.nih.gov/geo/) [1, 18, 20]. In all the data sets, patients were pre-defined as adenocarcinoma or neuroendocrine cancers based on NE-score, expression of NE markers like CHGA, SYP and pathological reports.

GSE 126078: The study was conducted among metastatic castration-resistant prostate cancer (mCRPC) patient specimens and patient-derived xenografts (PDX). The study identified subtypes with AR-low phenotype, amphicrine phenotype, double negative (AR-ve/NE-ve) phenotype and classical AR-ve/NE+ve phenotype through molecular profiling. In the deposited RNA-seq fastq files, cases were already identified with the sub-classes. We used those subclasses for our analysis.

GSE 66187: The study was performed among 50 mCRPC patients and 24 LuCaP prostate cancer-derived PDX to characterize neuroendocrine (NE) phenotype among these mCRPC specimens. The study involved IHC staining criteria for androgen receptor (AR) and prostate-specific antigen (PSA) expression, CHGA and SYP expression. Whole genome microarray, transcriptomics and IHC analysis were used to determine the NE phenotype.

GSE 137829: The study characterized the tumor-cell specific diversity from 6 mCRPC patients through single-cell RNA-Seq (scRNA-Seq) analysis. 4 of these mCRPC patients were identified as NE patients. SU2C-PCF: this is a multi-institutional prospective study, which involved a comprehensive genomic and transcriptomic profiling among 429 patients. The study integrated the findings of whole-exome, transcriptomics and histological analysis to provide a NE-like signature. All mCRPC patient cohorts who developed NE-like differentiation have received AR inhibitor treatments and represent treatment-refractory group. Based on the CHGA and SYP expression status, these entire cohorts have already subcategorized patients with adenocarcinoma and t-NEPC following treatment with second-generation AR signaling inhibition therapies and assigned with NE-score. The RNA-seq data was extracted from NCBI GEO using SRAtool Kit. The reads were then aligned to human reference genome (hg38 version) from UCSC genome browser and gene counts was quantified with HTSeq (v.0.9.1). Next, the raw counts were processed and normalized in DESeq2. Data processing was performed with the help of the Bioinformatics Core at UNMC.

### Cell culture

Cells were cultured in RPMI 1640 with 10% FBS in presence of penicillin-streptomycin antibiotics (0.1%). Upon reaching confluency, these cells were washed with 1X DPBS and trypsinized with 0.25% (w/v) Trypsin-EDTA to detach the cells from the plate. The cells were collected in equal volumes of complete medium to neutralize the effect of trypsin and further centrifuged at 1000 g for 5 min at room temperature. The pellet obtained from centrifugation was then resuspended in fresh complete media and plated in a T-75 flask and maintained at 37 °C and 5% CO2 in a tissue culture incubator. C4-2BER cells were cultured under continuous presence of 10uM Enzalutamide (MDV 3100, Selleckchem, S1250). Apalutamide-resistant cells (C4-2BAR) were generated from LNCaP C4-2B (which was a kind gift from Prof. Allen Gao) by culturing under 20 uM Apalutamide (ARN-509, Selleckchem, S2840) for 7–8 weeks. The murine syngeneic cell line (Developed by Dr. Batra) was derived from 6 months old Hi-Myc/PTENfl/fl/ Trp53R172H/+/Pb-Cre4+ positive mice (mixed strain, Hi Myc from FVB whereas Probasin Cre and Trp53R172H are from C57BL/6 background). The cells had undergone differential trypsinization to obtain pure epithelial cells from the stromal population and were maintained in a modified medium containing DMEM, 10% FBS, Penicillin and streptomycin, bovine pituitary extract (25 µg/mL) (Sigma, P1167), recombinant human EGF 25 µg/mL (Thermo, PHG0311L), insulin (5 ng/mL) (Sigma, I0516) and DHT (10 nM) (Sigma, D-073-1ML) [22, 23]. NCI-H660 cell line has been purchased from ATCC and cultured in HITES medium supplemented with 5% FBS, 0.005 mg/ml Insulin, 0.01 mg/ml Transferrin (Sigma, T8158), 30 nM Sodium selenite (Sigma, S5261), 10 nM Hydrocortisone (Sigma, H6909), 10 nM beta-estradiol (Sigma, E2758), extra 2 mM l-glutamine (Thermofisher, 25030081) (for final conc. of 4 mM) according to ATCC guidelines.

### Transient transfection

Cells were transfected with Pax5 siRNA (Dharmacon, ON-TARGETplus Human Pax5(5079) siRNA – SMARTpool, Catalogue #L-012241-00-0005) using TransIT-X2 Transfection Reagent (Madison, WI, Mirus, MIR6000) according to manufacturer’s protocol. A non-targeting siRNA (Dharmacon RNA Technologies, ON-TARGET plus, smart pool) was used as a control. A non-silencing shRNA (doxycycline inducible) was used as a control (Horizon Discovery Catalog ID:RHS4743). Cells were seeded at a density of 0.1×10^6^ cells in a 6-well plate. At 60-70% confluency, cells were transfected with specific siRNA (25 nM) or 2 different shRNAs. shRNA transfection is performed in a doxycycline inducible manner. Transfected cells were incubated for 24-48 h at 37°C and 5% CO_2_ in tissue culture incubator. Cells were transfected with Pax5 overexpression plasmid [Origene, PAX5 (NM_016734) Human Tagged ORF Clone – RC222785] for 18-24 h.

**Table:** 

shRNA	Clone Id	Mature antisense sequence
shPax5-1	V3THS_321775	TGATGAGCAAGTTCCACTA
shPax5-2	V3THS_321780	GTCCTGTCCTGCTGGTCCG

### RNA extraction

Total RNA was isolated by adding 1 mL of TRIZOL Reagent (ThermoFisher Scientific, CA) and allowed to stand for 5 min at room temperature. RNA isolation was performed with RNeasy Mini Kit (Qiagen, Germantown, MD) according to manufacturer’s protocol. The RNA pellet was dissolved in UltraPureDNAse and RNAse free water (Life Technologies, 10977-015). The concentration and quality of the RNA were analyzed using Nanodrop Spectrophotometer.

### Quantitative RT-PCR

1 µg RNA was used to synthesize cDNA with Transcriptor First strand cDNA synthesis kit (Roche Diagnostics Corporation) according to the manufacturer’s instructions. For real-time PCR, cDNA (50 ng) was used. PCR was performed in duplicates in 25 μl volume as described before [24]. 36B4 rRNA was used as internal control for normalization. The list of the primers used in this study is listed in the following table.

**Table:** 

Gene	Forward	Reverse
36B4	ATGCAGCAGATCCGCATGT	TCATGGTGTTCTTGCCCATCA
CHGA	TGTCCTGGCTCTTCTGCTCT	CAACGATGCATTTCATCACC
SYP	GATGTGAAGATGGCCACAGA	TCAGCTCCTTGCATGTGTTC
Pax5	GGCTCGTCGTACTCCATCA	GCACCGGAGACTCCTGAATA
Pbx1	CAAGCTAACTCGCCCTCAAC	CTGCACGCTCATGAACAAAT
NFASC	GACGAGCCGCTCTATATTGG	ACCAGGGCAGTTACACGTGT
JAG1	GGTGCGGTATATTTCCTCCA	TCCCGTGAAGCCTTTGTTAC
SMARCA4	GACAGTGAAGGCGAGGAGAG	CACTTTGACGGACCGAGATT
KIF9	GGGGGCAACTGAGAATTACA	GGCGTTCTTCGATCATCCTA
GRID1	GCTCCTCCTACACAGCCAAC	TGGACAGGTCCTGGAAAGTC
DPAGT1	GCGGTGCTGTTTTCCTTATC	GGGAATGCCTTACACTGCTC
NrCAM	CCCTGATTCTCTTCCTGTGC	CCCTGATTCTCTTCCTGTGC
TET2	ATTCTCGATTGTCTTCTCTAGTGAG	CATGTTTGGACTTCTGTGCTC
RB1	CTCTCACCTCCCATGTTGCT	GGTGTTCGAGGTGAACCATT
Sox2	CAAGATGCACAACTCGGAGA	GCTTAGCCTCGTCGATGAAC

### Western Blot

Cells were lysed with ice-cold CHAPS buffer (0.3% CHAPS, 40 mM HEPES pH 7.4, 10 mM β-glycerophosphate, 10 mM sodium pyrophosphate, 2 mM EDTA) having a combination of protease inhibitors, 10 μg/μL Leupeptin, 10 μg/mL Aprotinin, 1 mM PMSF and Halt protease. Cells were scrapped and lysed with 26G. The lysate was collected after being centrifuged at 13,500 RPM for five mins. The pellet was discarded, and the supernatant was used for protein analysis. Total protein estimation was carried out with Bradford reagent and the samples were prepared by the addition of SDS sample buffer containing β-mercaptoethanol and denatured at 95 °C for 5 min. The denatured samples were run on a precast 4–20% Mini-PROTEAN® TGX™ Gel (BioRad) and transferred onto a PVDF membrane (Life Technologies). The membrane was blocked in 3% bovine serum albumin (BSA) in 1X TBST (1X Tris Buffered Saline, 0.1% Tween-20) for at least 45 min. Primary antibody was diluted in blocking buffer and incubated with membrane overnight at 4 °C with continuous shaking at low speed. On the next day, the membrane was washed with 1X TBST for 3 times for 10 min and incubated in appropriate dilution of secondary antibody conjugated with HRP for 1 h in 1X TBST with continuous shaking at low speed at room temperature. Following this, the membranes were washed in 1X TBST for 6 times for 10 min each wash to remove the excess secondary antibodies. The protein bands were detected using a combination dilution of SuperSignal™ West Femto Maximum Sensitivity Substrate and SuperSignal™ Pico Maximum Sensitivity Substrate captured on an X-ray film.

**Table:** 

Antibody (host)	Dilution	Manufacturer
Pax5 (anti-Rabbit)	1:1000	Cell Signaling, 8970S
	1:1000	Abcam, ab109443
HSC70 (anti-mouse)	1:3000	Santa cruz, B-6, sc-729
Rho-GDI (anti-Rabbit)	1:3000	Cell Signaling, 2564S
Acetyl-Histone H3 (Lys 9) (anti-Rabbit)	1:1000	Cell Signaling, 9649S
Acetyl-Histone H3 (Lys 18) (anti-Rabbit)	1:1000	Cell Signaling, 13998S
Acetyl-Histone H3 (Lys 27) (anti-Rabbit)	1:1000	Cell Signaling, 8173
Histone H3 (anti-Rabbit)	1:1000	Cell Signaling, 9715S
AR (anti-Rabbit)	1:2000	Cell Signaling, 5153S
NCAM1 (anti-Rabbit)	1:1000	Cell Signaling, 99746S
TET2 (anti-Rabbit)	1:1000	Cell Signaling, 18950S
Pbx1 (anti-Rabbit)	1:1000	Genetex, GTX113242
GAPDH (anti-Rabbit)	1:3000	Cell Signaling, 2118L
DNMT1 (anti-Rabbit)	1:1000	Cell Signaling, 5032S
goat anti-rabbit IgG-HRP	1:10000	Invitrogen, 65-6120
goat anti-mouse IgG-HRP	1:8000	Invitrogen, 62-6520
Phospho-Akt S-473	1:2000	Cell Signaling, 4060S
Total Akt1	1:1000	Cell Signaling, 2967S
Phospho-EGFR	1:1000	Cell Signaling, 1068S
EGFR	1:1000	Cell Signaling, 4267S

### LuCaP and patient TMA for IHC analysis

LuCaP and mCRPC tumor microarrays were available commercially. These microarrays were obtained from the Prostate Cancer Biorepository Network (PCBN; LuCaP TMA number 90 A, B, C, D; mCRPC TMA number 92 A, B, C, and D). mCRPC TMA 92 contained tumor cores from multiple metastatic sites of 45mCRPC patients with known clinical diagnosis. A total of 15 out of 45 patients were clinically diagnosed with NE-like PCa. All the neuroendocrine patients were received ARSI therapies including abiraterone, enzalutamide and darolutamide at CRPC stage. Once their cancer progressed to neuroendocrine subtype, they received platinum therapies. Before preparation of TMA, further the cancer was validated for expression of AR, NKX3-1 and Syp. All the core were Syp positive for neuroendocrine patients’ tissues. Pax5 expression in these TMA slides was evaluated by IHC. As Pax5 expression is specific to NE-like cells, the IHC was calculated based on positive expression of Pax5. Here we calculated whether patients’ tissue was positive for Pax5 expression and correlated with their disease stage. For validation of Pax5 expression among t-NEPC patient cohorts, GSE126078, GSE 66187, Beltran 2016, and Stand Up to Cancer/Prostate Cancer Foundation) International Prostate Cancer Dream Team consortium, prospective clinical trial (identifier: NCT02432001) patient cohorts containing primary, mCRPC and NE-like PCa patient data were analyzed in-silico for Pax5 expression.

### Immunohistochemistry

Immunohistochemical analyses were conducted on formalin-fixed paraffin-embedded patient TMA as described before [24]. Antibodies used were provided in the table below. Tissue slides were scanned in UNMC Tissue Sciences Core Facility. Antigen retrieval was performed by heat induced epitope retrieval at 95 °C using Dako antigen retrieval solution (pH 9).

TMA (Both LuCaP and mCRPC Patient) (4 mm thick) were initially kept on heat block at 56 °C for 1 h to melt the paraffin. To completely dissolve the paraffin, the slides were sequentially passage through xylene for 30 min, and then 100% ethanol for 15 min. Next, the tissues were rehydrated by passing them through 95%, 90%, 80%, 75%, 50%, and 20% ethanol for 5 min each, and finally, it was immersed in double distilled water for 10 min. Antigen retrieval was carried out by heat induced epitope retrieval at 95 °C water bath using Dako antigen retrieval solution (pH 9). The antigen unmasking solution was initially preheated in the microwave until boiling and equilibrate them at a desire temperature (95 °C) for 30 min before put the slides. Slides were then immersed into this heated antigen retrieval solution for 30 min. Next, the slides were gradually cooldown at room temperature (leaving the slide in solution at room temperature) and washed with double distilled water. After that cellular peroxidase activity was inhibited by immersing the slides in 3% hydrogen peroxide (H2O2) in methanol for 10 min at room temperature. Before addition of antibody, slides were then blocked with 1% BSA + 0.2% saponin in TBST at room temperature for 1 h and then incubated overnight at 4 °C with primary antibody in the blocking buffer. Next day, after washing the primary antibody in TBST (3 times × 10 min), biotinylated secondary antibody (IHC) at desire concentration was added for 1 hr at room temperature. Next, after washing the secondary antibody, slides were then incubated with avidin–biotin complex (Reagent A and Reagent B) for 30 min at room temperature, following the manufacturer’s instructions. After that substrate diaminobenzidine solution (ImmPACT DAB) was added to the slides as a substrate for peroxidase until the desired staining intensity was developed. Hematoxylin was used as a counter staining. Finally, slides were dehydrated with increasing concentration of xylene and mounted with Permount with glass cover slips. All then slides were next digitally scanned. We have calculated the positive staining for Pax5 in the tissue cohort. The number of Pax5-positive samples was then correlated with the disease state of the patient or PDX.

**Table:** 

Antibody (host)	Dilution	Manufacturer
Pax5 [EPR3730 (2)] (anti-Rabbit)	1:250	Abcam, ab109443

### Immunofluorescence

Cells were transfected with siPax5 or shPax5 (2 independent clones as described before) and Scramble as previously described. For rescue experiments, Pax5 knockdown cells (siPax5 or shPax5 transfected cells) were transfected with Pax5 overexpression plasmid. Cells were then incubated for 24-h at 37 °C and 5% CO2 tissue culture incubator. After 24–36 h incubation, cells were washed twice with 1X DPBS and fixed with 4% paraformaldehyde for 15–20 min. Cells were blocked in 0.2% saponin in 1% BSA prepared in 1X TBST and incubated overnight with respective primary antibodies diluted in blocking buffer. Cells were washed 2 times with 1X TBST for 5 min each wash. Cells were incubated with fluorophore-conjugated anti-rabbit and anti-mouse secondary antibodies for 30 min, respectively. Cells were next washed in 1X TBST and mounted with Vectashield mounting media containing DAPI (Vector Laboratories, H1200), and photomicrographs were captured using the confocal microscope.

**Table:** 

Antibody (host)	Dilution	Manufacturer
Pax5 [EPR3730(2)] (anti-Rabbit)	1:250	Abcam, ab109443
Fluorescein Phalloidin	1:200	Invitrogen™, F432
NCAM1 (anti-Rabbit)	1:100	Cell Signaling, 99746T
Phospho-Akt S-473	1:100	Cell Signaling, 4060S
Phospho-EGFR	1:100	Cell Signaling, 1068S

All images taken in confocal microscopy were captured using Zeiss LSM 800 Confocal Laser Scanning Microscope (equipped with 4 different lasers) in UNMC confocal microscopy Core Facility. Analysis of captured images and their quantification were done using Zeiss Zen 2010 software and ImageJ software respectively. The graphical illustrations were made using GraphPad Prism 8 software.

To determine surface intensity of fluorescence image, we have used Fiji software. After selecting the cell surface, we first clear out the background. Then we performed auto threshold adjustment of images. Using this threshold adjusted image, average fluorescence integrated density was calculated. This fluorescence intensity was subtracted from the background intensity to get the corrected average integrated density per cell. Total 10 cell was counted per filed and 5 field was taken for analysis of each replicate experiments. Experiments has been repeated atleast three times. For neurite analysis, no of neurite/cell was calculated. A total of 150 cells were calculated/experiments.

### 5-Azacytidine assay

Cells were seeded at a density of 0.1 × 10^6^ on a 6-well plate. Cells were exposed to 5-azacytidine (at a dose of 0.1 µM) and incubated for 6 days in tissue culture incubator. Cells were treated with 5 μM Enzalutamide (AR inhibitor) to block the nuclear transport of AR. Cells were incubated in tissue culture incubator for 6 days. Cells were next collected and lysed.

### Tet-activity inhibition

Cells were treated with Bobcat 339 (Selleckchem.com, catalog no. S6682) at a dose of 50 μM (IC_50_ = 33 μM for Tet1 and 73 μM for Tet2) and incubated for 18–24 h in tissue culture incubator. After incubation, cells were subjected to ChIP assay using 5hmC and PBX1 antibodies respectively.

### Cell death assay with propidium iodide staining

Cells were transfected with a scramble and Pax5 siRNA as mentioned earlier. Pax5 knockdown cells were transfected with Pax5 overexpression plasmid (rescue). Cells were then incubated for 24 h at 37°C and 5%CO2 tissue culture incubator. Cells were next treated with Docetaxel at various concentrations (2 nM for C4-2BER and 10 nM for DKD, respectively) and kept incubated for the next 18–24 h. After incubation, cells were washed gently with 1XDPBS and incubated with propidium iodide (PI) (1:2000 dilution in PBS) for 10–15 min. Hoechst (1:2000 dilution in PBS) was used to stain the nucleus. Images were captured under 10X in UNMC confocal core facility.

### Surface biotinylation assay

Cells were transfected with doxycycline-inducible Pax5 shRNA for 48 h. Control and shRNA transfected cells were carried out for labeling cell surface with biotin under cold conditions following the manufacturer’s guidelines (Pierce Cell Surface Protein Isolation Kit, Thermo Scientific #89881). The biotin-labeled proteins were eluted and analyzed by western blot.

### RNA-Seq

RNA Seq was performed for LNCaP C4-2, LNCaP C4-2 DKD, LNCaP C4-2B, LNCaP C4-2B-ER in triplicate condition. In addition, we have carried out RNA-Seq following depletion of Pax5 by siRNA in DKD and C4-2BER cells. RNA from cells was isolated using the RNeasy Mini Kit (Qiagen, Germantown, MD) following the manufacturer’s guidelines. The quality and integrity of RNA were confirmed using the Agilent Bio-analyzer. Paired end run of RNA libraries was carried out with Illumina NextSeq 500. Sequences were next aligned to the human reference genome (hg38 version) from the UCSC genome browser. Data analysis was performed with the help of the Bioinformatics Core at UNMC. Estimation of RNA abundance was carried out with feature Counts from the Sub-read package version 1.6.3. Downstream analyses were performed with the DESEQ2 R package version 1.18.1 [25]. A Principal component analysis (PCA) was performed using https://biit.cs.ut.ee/clustvis/. Differentially expressed genes (DEGs) were identified by pairwise comparisons with the DESEQ2 package (v.1.12.3). Genes were retained as differentially expressed when the fold-change (FC was >2 or <-2). The raw data for DKD vs C4-2 RNA-Seq can be accessed from GSE202299.

STAR was used to map RNA-seq paired-end reads to the hg38 human reference genome [26]. StringTie was used to create count matrices and perform Transcript Per Million (TPM) normalization [27]. DESeq2 was used to calculate differential gene expression and perform clustering of samples from each replicate. Genes with a padj value < 0.05 and a log2 fold change > = 1 or > = -1 were considered significantly differentially-expressed. EnhancedVolcano was used to generate a volcano plot comparing RNA expression between C4-2B and C4-2BER (https://github.com/kevinblighe/EnhancedVolcano). Gene ontology analysis of differentially expressed genes was performed using the web-based EnrichR gene-list enrichment analysis too [28]l. Gene Set Enrichment Analysis (GSEA) on the differentially expressed genes was performed using Webgestalt [29]. The Canonical Pathways analyses were generated through the use of Qiagen Ingenuity Pathway Analysis [30].

### ATAC-Seq

Cells viability and cell counting were performed using an automated cell counter (Biorad). Cells were centrifuged at 500 g before proceeding with nuclei extraction. Nuclei were then isolated on an iodixanol gradient (The lysis was performed using 0.4% Igepal CA-630, 0.4% Tween 20 for 3 min) and counted after addition of trypan blue using automated cell counter (Biorad). 50,000 counted nuclei were transferred to a new tube and were centrifuged at 500×*g* before proceeding with the transposition reaction. Isolated nuclei were lysed and transposed for 30 min at 37 °C using the prokaryotic Tn5 transposase system (Nextera DNA library kit, Illumina, FC-121–1030). Transposed DNA was then purified on Diapure columns (Diagenode, C03040001). After their amplification, the libraries were size selected and purified using Agencourt AMPure XP (Beckman Coulter) and quantified using Qubit dsDNA HS Assay Kit (Thermo Fisher Scientific, Q32854). ATAC-Seq libraries were generated and then re-sequenced in paired-end mode 50 base pairs (PE50) on an Illumina NovaSeq 6000, running NovaSeq Control Software version 1.6.0. FastQC was used to determine the quality control of the sequenced reads. Nextera adapters were trimmed from the reads using cutadapt (https://cutadapt.readthedocs.io/en/stable/) -a CTGTCTCTTATACACATCT -A CTGTCTCTTATACACATCT -m 20 -q 20 –pair-filter=both. Trimmed reads were aligned to the human reference genome (hg38 version) using Bowtie2 -X 2000 –no-mixed –no-discordant [31]. Reads were quality filtered based on mapQ ≥ 10 using samtools [32] and duplicate reads were removed using picard (https://broadinstitute.github.io/picard/). After deduplication, biological replicates were merged and peaks were called using macs2 –nomodel –shift 100 –extsize 200. Bigwig ATAC-seq signal tracks were created from the merged mapped reads using deeptools with BPM normalization [33, 34].

### Differential promoter activity analysis

BEDTools Intersect Bed was used to find ATAC-seq peaks overlapping differential H3k27ac and H3k18ac peaks. ATAC-seq, H3K27ac ChIP-seq, and H3K18ac ChIP-seq signal was plotted across ATAC-seq peaks with differential or unchanged H3K28ac and H3K18ac ChIP-seq peaks [35]. The ATAC-seq and ChIP-seq heatmaps were generated using deeptools. To determine differential transcription factor motif activity in C4-2BER, ATAC-seq peaks overlapping increased H3K27ac and H3K18ac peaks within -2000 bp and +500 bp of promoters was used for motif analysis. BEDTools getfasta was used to acquire the fasta sequence of ATAC-seq peaks with differential H3K18ac/H3K27ac and used as input for subsequent meme-ChIP and tomtom analysis [36–38]. The human mononucleotide HOCOMOCO v11 motif collection was used for meme-ChIP and tomtom motif enrichment [39]. Finally, the total list of enriched motifs from meme-ChIP and tomtom was further filtered for transcription factors upregulated in C4-2BER with a log2 fold change >1 and a padj < 0.05. The Broad Institute Integrative Genomics Viewer was used to visualize ATAC-seq and ChIP-seq signal [40].

#### Transfac gene regulation database

The database (http://genexplain.com/transfac/) has been accessed and transcription factor selection for Pax5 promoter site was performed through MATCH tool as described [41].

### ChIP-seq and ChIP-qPCR

ChIP assay was performed with Magnify^TM^ ChIP System (Invitrogen, Life Technologies, 49-2024) according to manufacturer’s guidelines. Cells plated on 100 mm culture dish were washed twice with 1X DPS and scraped off. Cells were centrifuged at 1000 rpm for 5 min at room temperature. Cells were then fixed with 37% formaldehyde for DNA-protein crosslinking and incubated for 10 min at room temperature. The crosslinking reaction was stopped by adding 1.25 M glycine for 10 min at room temperature. The cells were next centrifuged at 200 g for 10 min ta 4°C. The pellet was further lysed for nuclear and cytoplasmic separation. The supernatant was discarded, and the pellet was lysed in hypotonic Buffer A (10 mM HEPES pH 7.8, 10 mM KCl, 2 mM MgCl2, 0.1 mM EDTA) with 0.5 M DTT and 1% each of 0.1 M PMSF, 10 µg/µl Leupeptine, 10 mg/ml Aprotinin protease inhibitors and Halt phosphatase inhibitor and incubated on ice for 17 min. After incubation, the samples were vortexed mildly, and 10% NP-40 was added with a further incubation of 4–5 min. This was further centrifuged at 1000 rpm for 5 min at 4 °C. The supernatant containing the cytosolic contents was discarded and the nuclear pellet was further processed to yield quality DNA as per the manufacturer’s guidelines. 2–10 ng of purified DNA was sent for sequencing. Sequencing was performed on an Illumina Novaseq 6000, running NovaSeq Control Software 1.6.0. Quality control of sequencing reads was performed using FastQC. Reads were aligned to the reference genome (GRCh38/hg38) obtained from the UCSC genome browser using bowtie2 version 2.3.4.1 [31]. Samples were filtered for regions blacklisted by the ENCODE project [42]. Reads were quality filtered based on mapQ ≥ 10, and duplicate reads were removed using picard (https://broadinstitute.github.io/picard/). After deduplication, biological replicates were merged and peaks were called using macs2 [43]. Differential ChIP-seq peaks were determined using manorm [44]. Bigwig ChIP-seq signal tracks were created from the merged mapped reads using deeptools with BPM normalization [33]

For ChIP-qPCR analysis, we have developed the primers by analyzing the ChIP-Seq peaks of PBX1 [45] or Pax5 [46] from Cistrome db.

**Table:** 

ChIP grade antibody (host)	Manufacturer
Acetyl-Histone H3 (Lys 18) (anti-Rabbit)	Cell Signaling, 13998S
Acetyl-Histone H3 (Lys 27) (anti-Rabbit)	Cell Signaling, 8173S

After isolating the ChIP DNA (as mentioned above), qPCR was carried out with the following primers. The ChIP primers sequences are mentioned as follows.

**Table:** 

ChIP grade antibody (host)	Manufacturer
Pbx1 (Rabbit)	Thermo fisher Scientific, #PA5-17223
5hmC (Rabbit)	Active Motif, #39069
Pax5 [EPR3730(2)] (anti-Rabbit)	Abcam, ab109443

**Table:** 

Pbx1_ChIP_Region	Forward	Reverse
Primer 1	GCGAAATCTGCTCAGTGGATA	CCTAGGGGGAAGAGCCTAGA
Primer 2	CAACAACAAAACACCAACA	CCTAGGGGGAAGAGCCTA
5hmC ChIP_promoter CpG	ATAGAAGGTGCGGCTGGAA	CTACGGGAAGGGGCAGAC

**Table:** 

Pax5_ChIP_Region	Forward	Reverse
JAG1_Primer 1	AACCATGAAATAGACTCTCGG	GTTTCTCCAACCACATACAGA
JAG1_Primer 2	AGGCACCACTGAAAATGT	CCAACCACATACAGAAAAACA
SMARCA4_Primer 1	AGAAAAATCAAGCCAGGATA	ACCTTCTCCTTTTCCCAGAA
SMARCA4_Primer 2	GGATAGAGAGGAAGGAACGG	TACTCTGGCTCATGCAGG
KIF9_Primer 1	TCCGCCGAAGTCTTTCTAGA	TGGCGGAAATGAAGTCC
KIF9_Primer 2	TCACCTTTTCATCTCAAGGC	GACTCTGAGACCCCAAAG
DPAGT1_Primer 1	CTCTCGGTGATTCTACTCTTGA	CAACCATTACTGCGGAAGG
DPAGT1_Primer 2	GGGGCAGAACATAGGTT	CTTCAGGTAACGGGCAA
GRID1_Primer 1	AGAAACCACATCCTGCATT	TATTTCTGTCTGGACATGG
GRID1_Primer 2	CATGTGATGCATCACATAAT	TCTTGTTTTATTTTCCATGG
NrCAM	GGAACTTCATGACAGAAATAAA	CTCCCTCTCAAAAAACAAAC
NFASC	ATTTGACCCCGTTACCC	ACTTTGCGGTGGATCTA
PGM5 P1 (site 1)	AAACCCAGACTGACAAGGAG	CCTCAAGATCCAGTGCCAAA
PGM5 P2 (site 1)	CACAGTACATGAGGTGGCA	ATAATGCACAGACCACACCA

### EPIC methylation array

The Infinium MethylationEPIC BeadChip assay, a genome-wide DNA methylation analysis technique has been performed with C4-2 and DKD cells. This array-based assay uses bisulfite converted DNA and Illumina® technology to quantitatively detect the CpG island methylation level throughout the genome at a resolution of single nucleotide bases.

Deamination of DNA is performed with the EZ-96 DNA Methylation Kit (Zymo Research) according to Illumina’s guidelines. Array Scan Infinium Control BeadChips have been used which are equipped with a set of internal control probes. These control probes are used for identification of test samples with different data characteristics based on threshold parameters. These controls are also evaluated as per relative intensities. are The EPIC Array analysis has been done with GenomeStudio® Software 2011.1, Methylation Module v1.9 following the Illumina Methylation Module user guidelines (Controls Dashboard).

### Statistical analysis

All the graphical illustrations and statistical tests were performed using GraphPad Prism 8 software (GraphPad software, Inc.). All data reported in graphs are expressed as mean ± standard deviation (SD), unless otherwise mentioned, and were compared using a standard two-tailed unpaired t test unless otherwise mentioned. Statistical significance of data was assessed using non-parametric Student’s *t* test, one-way ANOVA. Results with *P*-value ≤ 0.05 were considered statistically significant. All experiments were performed in 3 independent replicates.

## Results

### NE-like transformation adapts to a specific gene signature

To understand how neuroendocrine prostate cancer functionally differs from adenocarcinoma, we analyzed published RNA-seq data derived from patient tissues (GSE126078, GSE66187, SU2C [20]). We used their pre-defined classifications, which incorporate an NE-score, marker expression, and pathology to compare patients consistent with t-NEPC to that of adenocarcinoma (details in materials and methods). Next, differentially expressed genes were analyzed through Gene Set Enrichment Analysis (GSEA), which demonstrated that pathways related to secretion, synapse assembly, and neuronal signaling (Fig. S1A) are preferentially upregulated in the t-NEPC cases.

To investigate the significance of t-NEPC-specific neuronal gene signatures, we have developed various cell lines, which can replicate neuroendocrine characteristics. Earlier we showed that these cell lines express neuroendocrine markers such as synaptophysin (Syp) and chromogranin (CHGA) and lack AR expression (Fig. S1C from ref. [19]. They are referred to as C4-2BER (by continuous exposure of adenocarcinoma cell line C4-2B to enzalutamide) and DKD (depleting RB1 along with TP53 from adenocarcinoma cell line LNCaP C4-2) respectively [19]. We previously demonstrated that enzalutamide-resistant C4-2BER cells lose the expression of RB1 and TP53, which are the main factors behind the NE-like transition [19].

**Fig. 1:**
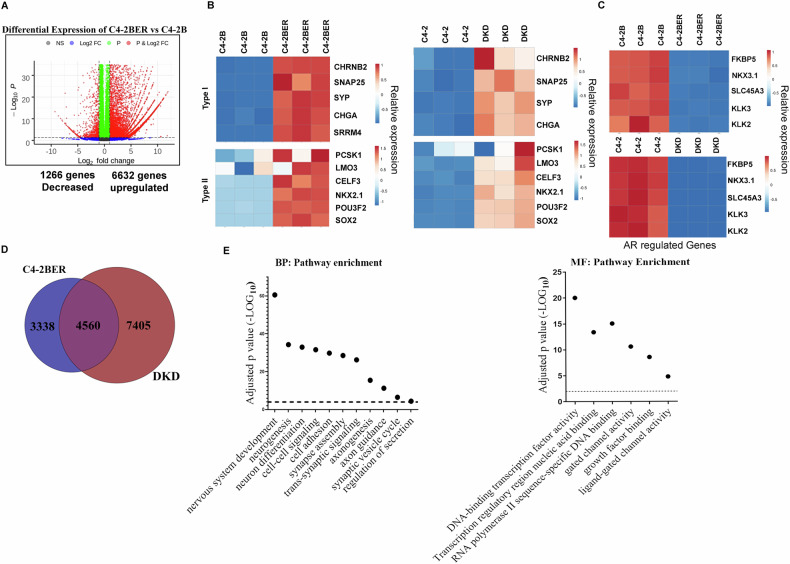
Neuroendocrine differentiation carries neuronal signature. **A** Differential RNA-expression between C4-2BER and C4-2B cells. Red indicates genes that meet a *p*-value cutoff of <0.05 and have fold change ≥2. **B** Comparative gene expression study between the adeno (C4-2B and C4-2 in triplicates) and NE-like cells (C4-2BER and DKD in triplicates), respectively. Type I gene-set represents the genes regulated by REST repressors and Type II indicates the transcriptional regulators involved in neuroendocrine pathways. Expression of RNA was analyzed following the RNA-seq in all these cell lines. **C** Comparative analysis of AR regulated genes in similar adeno and neuroendocrine cell lines. **D** Venn Diagram represents the common differentially expressed genes in two different neuroendocrine-like cell lines. **E** Using common differentially enriched gene-sets, pathway analysis was carried out with gProfiler. BP biological pathway, MF molecular pathway. Scattered plots represent the differentially enriched pathways associated with neuroendocrine transformation.

To understand whether the cell lines we developed feature the t-NEPC like characteristics, we have initially carried out RNA-seq (in triplicate) between C4-2B and C4-2BER. Our results suggested that 6,632 genes are significantly upregulated and 1266 were downregulated in C4-2BER as compared to C4-2B (Fig. 1A). Further studies revealed that an increase in REST-repressed neuroendocrine gene signatures (Type I genes such as CHGA, SYP, SNAP25, CHRNB2 and SRRM4) and transcriptional regulators for t-NEPC differentiation (Type II gene sets such as Sox2, NKX2.1, POU3F2, etc.) (Fig. 1B, first panel) is highly upregulated in C4-2BER as compared to its parental adenocarcinoma cell line C4-2B. These neuroendocrine specific gene-expression were comparable with t-NEPC patient gene expression data sets reported earlier [1, 18]. Similar results were also observed when we compared the RNA-seq between C4-2 (adenocarcinoma cell line) and DKD (t-NEPC) (Fig. 1B, second panel) [we have reported the RNA-seq in our earlier publication [19]]. Moreover, our derived neuroendocrine cell lines also express neuroendocrine-related markers [19] such as beta III tubulin [19] and NeuN (Fig. S1B). On the contrary, AR expression (Fig. S1C) as well as AR driven gene signature such as expression of KLK3, or Nkx3.1 decreased in C4-2BER and DKD (representing t-NEPC) cells as compared to adenocarcinoma C4-2B and C4-2 cells respectively (Fig. 1C). Further, using RT-PCR, we validated that other AR regulated genes such as KLK2 and TMPRSS2 are also down-regulated in C4-2BER, DKD and NCI-H660 (a representative de novo neuroendocrine cell line from ATCC), and some of the features of t-NEPC have high similarity with this de novo neuroendocrine cancer cell line (Fig. S1D). These results indicated that our developed cell lines have acquired NE-like features.

To determine whether our NE-like cell lines exhibited neuronal features comparable to those seen in t-NEPC patients (Fig. S1A), we first examined the common genes that were differentially regulated in both C4-2BER and DKD cell lines. We identified 4,560 common differentially expressed genes between C4-2B vs C4-2BER and C4-2 vs DKD respectively (Fig. 1D), which were used for pathway analysis using g-Profiler [47], as well as gene ontology (GO) over representation analysis (ORA) and IPA (Fig. 1E and S1E-H). Interestingly, pathway analysis using gene expression profile revealed that, similar to t-NEPC patients (Fig. S1A), C4-2BER and DKD show enrichment of neuronal pathways (Fig. 1E and S1E-H) related to neuronal adhesion, secretion, exocytosis, and axonal guidance. Overall, these findings indicate that our NE-like cells acquired neuronal behavior, which could be important for their AR-independent growth and survival.

Morphologically, our developed NE-like cell lines showed neurite-like protrusion while growing in 2D attachment culture [(Fig. S1C from Islam et al.) and ref. [19]]. Interestingly, these derived cell lines grow partly in attachment and partly in suspension (similar to NCI-H660 or SCLC cell lines) and these suspended cells form strong cellular aggregates (Fig. S1I). Reports suggest that during small-cell neuroendocrine transformation, cells form spherical aggregates and appear as crowded morphology [48–50]. Whether such small cell characteristics are associated with enhanced therapy-resistance, has not been studied. Earlier, we showed that, similar to neuroendocrine-like cancers, DKD and C4-2BER cells are highly resistant to taxane-based chemotherapies such as docetaxel as compared to its parental adenocarcinoma lines C4-2 and C4-2B [19]. Here we predicted that neuronal adaptation is crucial for establishing strong cellular contact, thereby displaying a crowded phenotype which is associated with taxane resistance in clinically aggressive t-NEPC. Below, we will investigate how these t-NEPC cells developed neuronal-like traits and whether any key transcriptional events are associated with such morphological features.

### Increased chromatin accessibility and histone acetylation induce transcriptional activation during NE-like transformation

**Fig. 2:**
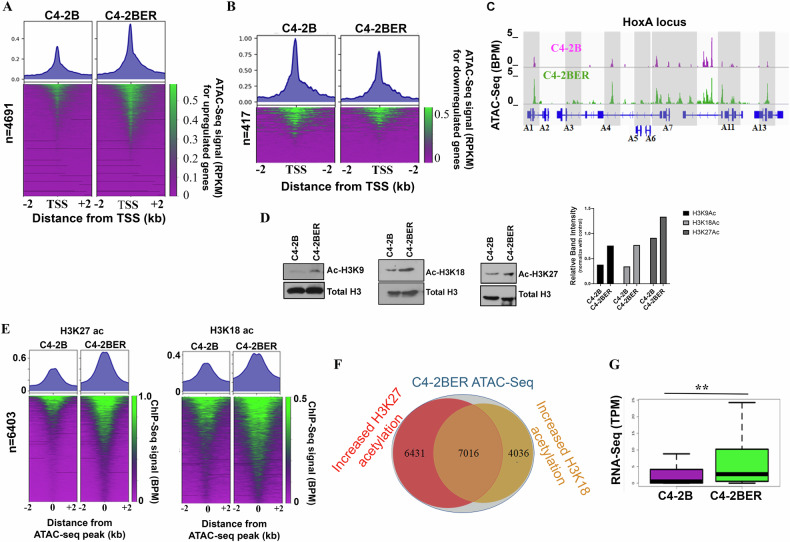
Differential chromatin accessibility in Neuroendocrine differentiation. **A** Heatmap showing comparison of ATAC-Seq signals (BPM normalized) between C4-2B and C4-2BER for differentially upregulated genes in C4-2BER. **B** Heatmap showing comparison of ATAC-Seq signals (BPM normalized) between C4-2B and C4-2BER for differentially downregulated genes in C4-2BER. **C** IGV browser showing differential ATAC seq signals (BPM) for Hox A gene locus in C4-2B and C4-2BER. **D** Immunoblot showing histone acetylation levels of H3K9, H3K18, and H3K27 in C4-2B and C4-2BER, respectively. Quantification of the bands are shown in a bar graph. **E** Heatmap showing a comparison of ChIP-Seq signals (BPM normalized) for H3K27ac and H3K18ac in C4-2B and C4-2BER at differentially active loci. **F** Venn diagram showing overlap of differential ChIP-Seq (H3K18ac and H3K27ac) peaks that occur at C4-2BER ATAC-seq peaks. **G** RNA-seq expression levels (TPM) for genes near differentially active chromatin loci from Fig. 2F. **p-value < 0.001 wilcoxon rank-sum test.

Reports indicate that during NE-like transformation, adenocarcinoma cells undergo a series of chromatin modification and epigenetic alterations to enhance neuroendocrine-related gene expression [51, 52]. To understand whether our derived cell lines follow such characteristic changes in their DNA, we have carried out ATAC-Seq as well as histone ChIP-seq. Chromatin accessibility near promoter sites is an important component of transcriptional activity regulation in a cell [53]. To understand whether differential upregulation of gene expression can be explained by altered chromatin accessibility, we performed assays for transposase-accessible chromatin with sequencing (ATAC-seq) in C4-2BER and compared it with C4-2B. As ARSI resistance is the major factor driving NE-like transformation [2, 54], we investigated chromatin alteration during NE-differentiation in C4-2BER cells using high throughput sequencing and compared them to adenocarcinoma C4-2B cells. We examined ATAC-seq signal near proximal promoters (±2 kb of the transcription start sites [TSSs]) of upregulated genes defined by *p*-value < 0.05 and at least a 4-fold change in expression. Our ATAC-seq results display a marked increase in chromatin accessibility around upregulated genes’ TSSs in C4-2BER compared to C4-2B (Fig. 2A). In contrast, we see slightly decreased accessibility for downregulated genes in C4-2BER (Fig. 2B). This pattern of altered accessibility explains the potential of C4-2BER to maintain differential transcriptional activity during NE-like differentiation from adenocarcinoma. For example, increased chromatin accessibility is evident across the Hox A locus corresponding to the increased expression of Hox A genes (Figs. 2C and S2A). We detect similar accessibility changes near the TSS of genes that encode various cell adhesion genes including neuronal adhesion proteins, such as NCAM1, VCAN, CD40 which coincides with their higher expression in C4-2BER **(**Fig. S2B-C). Interestingly, earlier studies have shown that overexpression of Hox genes or NCAM1 is linked with either a NE-like transformation or the development of AR signaling inhibitor-resistant prostate cancer [11, 55–57].

To further explore what drives the chromatin accessibility for these t-NEPC gene signatures, we examined histone acetylation levels, which are commonly used as marks of transcriptionally active chromatin [58]. Our results indicate a global increase in acetylation of histone H3 at lysine 9, lysine 18 and lysine 27 in C4-2BER compared to its adenocarcinoma (Fig. 2D). Similarly, we detected increased H3K27Ac and H3K18Ac in DKD when compared to its parental line C4-2 (Fig. S2D). To evaluate the influence of acetylated histones on genome-wide chromatin accessibility, we performed chromatin immunoprecipitation with sequencing (ChIP-Seq) for H3K27Ac and H3K18Ac in C4-2BER and C4-2B respectively. Consistent with activated chromatin, we found significant increases in H3K27ac and H3K18ac at C4-2BER accessible regions compared to parental C4-2B (Fig. 2E). To decipher the link between accessible chromatin sites and active histone acetylation marks, we determined the overlap of increased H3K18Ac and H3K27Ac ChIP-Seq peaks at C4-2BER ATAC-seq peaks. Indeed, we identified 7,016 accessible regions that coincide with increases in both H3K27Ac and H3K18Ac (Fig. 2F). Examining our RNA-seq data, we found that these accessible regions correspond to increased expression of nearby genes such as CHGA, ASCL1 (Figs. 2G and S2E). Altogether, our findings suggest that a chromatin-based activation of putative promoters can explain the gene expression signature specific to t-NEPC compared to adenocarcinoma.

### Pax5 expression increases following neuroendocrine trans-differentiation

**Fig. 3:**
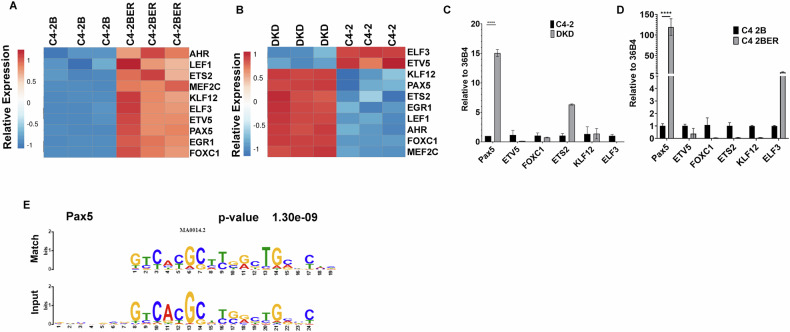
Differential transcription factor binding near newly accessible promoters. **A** Heatmap showing expression of transcription factors in A. C4-2B and C4-2BER cells (*N* = 3). **B** C4-2 and DKD cells (*N* = 3). **C**, **D** RT-PCR showing the relative gene expression of Pax5, ETV5, FOXC1, ETS2, KLF12, ELF3 in C4-2 vs DKD and C4-2B vs C4-2BER cells respectively. **E** Image showing the identified motif at differentially active loci compared to the Pax5 motif as determined by TOMTOM.

Chromatin accessibility governs the ability of transcription factors to bind to their target loci, thereby controlling the transcriptional output of a cell. To explore the preferential binding of potential transcriptional regulators near the differentially exposed gene promoters in t-NEPC, we selected the 7,016 peaks (Fig. 2F) that represent highly accessible promoters (from ATAC seq signal), with active histone marks H3K27ac and H3K18ac. These peaks correspond to promoters of transcriptionally active genes with increasing expression (Fig. 2G) in C4-2BER. Using MEME-ChIP in combination with TOMTOM motif comparison tool, we identified an array of transcription factor motifs in these highly accessible promoters of differentially upregulated genes in C4-2BER (Table S1). Based on P-value significance score, we further narrowed down to the top 10 transcription factors (Table S1). Further, we examined the expression of these TFs among t-NEPC patient cohorts, which showed consistent upregulation of Pax5, ETV5, and KLF12 (Fig. S3A). While analyzing these CRPC-adenocarcinoma and t-NEPC patients’ individual expression for Pax5, ETV5 and KLF12, we further found that both ETV5 and KLF12 expression vary among adenocarcinoma and t-NEPC patients, with sometimes showing higher expression in CRPC adenocarcinoma (Fig. S3B). However, among these, Pax5 showed consistently higher expression specific to t-NEPC patients (Fig. S3B). Additionally, Pax5 showed consistently high expression between our different cell line models (Fig. 3A, B) and has been associated with neuronal gene activation [59, 60]. Using RT-PCR, we have validated that compared to ETV5 or KLF12, Pax5 is highly expressed in DKD cells (Fig. 3C). Similarly, we have checked the KLF12, Pax5 and ETV5 expression in other cell lines (Fig. 3D and S3C, D). Our results indicates that Pax5 in consistently upregulated in NE-like cell lines. This data indicates that Pax5 expression is significantly associated with the neuroendocrine transformation.

Indeed, TOMTOM motif analysis of sites with increased chromatin accessibility (from ATAC-Seq) carrying H3K18ac and H3K27ac footprints within the TSS ± 1000 bp in C4-2BER reveals a motif (*P* = 1.30e-09) similar to the Pax5 recognition motif (Fig. 3E). We have tested Pax5 motif in our cell lines and found that its target genes such as TNC or DAB1, which are highly expressed in NE-like cells, have increased promoter chromatin accessibility and histone acetylation compared to adenocarcinoma cells (Figs. S3E and S3F). Together, these results suggest that increased accessibility and active chromatin marks at Pax5 binding sites near NE-specific gene promoters is associated with t-NEPC transformation.

**Fig. 4:**
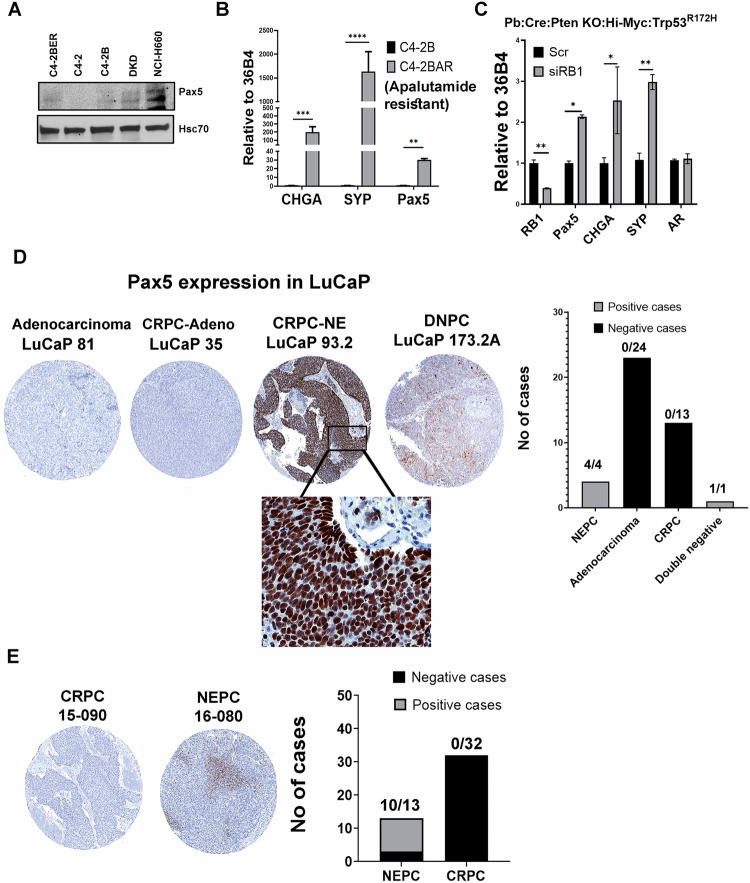
Expression of Pax5 in various cell types. **A** Immunoblot of comparative Pax5 Expression in C4-2, C4-2B, C4-2BER, DKD, and NCI-H660. HSC70 is the loading controls. Immunoblots are representative of *N* = 3 independent experiments. **B** RT-PCR showing RNA expression of Pax5 in C4-2B and C4-2BAR cells. Student’s *t* test representing statistical significance. ***P < 0.0001, **P < 0.001, and *P < 0.01. Error bars represent standard errors between N = 3 biological replicates. **C** Pax5 expression following the depletion of RB1 from the mouse cell line derived from the transgenic animal PB-Cre:High-Myc/Pten KO/Trp53^R172H^. **D** Pax5 was stained in LuCaP TMA. Representative IHC staining shows the expression of Pax5 in adenocarcinoma, CRPC and NE-like cancers. Black highlighted area showing Pax5 nuclear staining in ×40. Bar graph represents Pax5 positive and negative cases. Numbers above the bar indicate the number of Pax5 positive cases out of total number of cases. **E** Pax5 was stained in metastatic prostate TMA. Representative Pax5 expression by IHC is shown in the figure. Bar graph represents the number of Pax5 positive and negative cases. Numbers above the bar indicate the number of Pax5 positive cases out of total number of cases.

To understand whether Pax5 expression is specific to t-NEPC, we have analyzed both RNA and protein expression of Pax5 in our models and compare them with CRPC adenocarcinoma cell lines. t-NEPC shares very similar characteristics and gene expression profiles with de novo neuroendocrine cancers [61, 62]. Similar to our NE-like cell lines, de novo small-cell neuroendocrine cell line NCI H660 expresses all the classical NE-like markers with no AR expression [61]. Our results show that Pax5 is preferentially expressed in DKD, C4-2BER and NCI-H660 as compared to adenocarcinoma counterpart C4-2 and C4-2B respectively (Fig. 4A, Fig. S4A). Further, Pax5 expression was not upregulated in AR-null PC3 cell line (Fig. S4A). Further, using apalutamide (another ARSI) resistant NE-like C4-2B cells (known as C4-2BAR), we validated Pax5 expression (Fig. 4B). From the above evidence, our results indicates that Pax5 expression is mainly associated with neuroendocrine-like transformation.

Earlier, Ku et. al. showed that mice develop metastatic prostate cancer by genetically knocking out prostate-specific Trp53 and RB1 genes [63]. Some of these metastatic loci showed neuroendocrine differentiation and often presented with low luminal keratin with high NE marker expression (Krt8-low:Syp-high:AR-low). On the other hand, adenocarcinoma had higher luminal keratin and AR expression with low expression of NE-associated genes (represented as Krt8-high:Syp-low: AR-high). They studied the overall gene expression of various metastatic foci to identify the differential genetic signature associated with the development of metastatic neuroendocrine and adenocarcinoma (GSE90891). By re-analyzing these RNA-seq expression data, we found that Pax5 expression is higher in metastatic cancer with neuroendocrine differentiation (Fig. S4B). Moreover, we observed an increase in Pax5 expression in mouse-derived cell lines (Hi-Myc/PTEN^fl/fl^/ Trp53^R172H/+^/Pb-Cre4^+^ mice) following depletion of RB1 (Fig. 4C), which is also concurrent with high NE marker expression. Therefore, these results suggest that depletion of RB1 in TP53 knockout background induces NE-like characteristics, which is associated with Pax5 expression.

Finally, we validated Pax5 expression in tissue microarrays (TMAs) derived from prostate cancer patient-derived xenografts (PDX) (LuCaP series) and from metastatic CRPC (mCRPC) tissues obtained from Prostate Cancer Biorepository Network (PCBN). The detailed characterization of these PDXs have been described elsewhere [64]. The LuCaP TMA from PDX contain 42 patient tissues (24 are from adenocarcinoma, 13 from CRPC, 4 patients are from t-NEPC and one AR null, NE null prostate cancer group often referred as double negative stage) in triplicate. We validated the nuclear expression of Pax5 (Fig. 4D), in LuCaP PDX with t-NEPC. LuCaP derived from CRPC-adenocarcinoma stained negative for Pax5 expression, whereas only t-NEPC LuCaP PDXs (LuCaP 93, LuCaP 145.1 LuCaP 145.2, and LuCaP 173.1), stained positive for Pax5 expression. These results once again indicate that, overall, Pax5 expression is associated with NE-like transformation.

Similar to LuCaP models, we also analyzed Pax5 expression in the TMA derived from the metastatic PCa patients [Prostate Cancer Biorepository Network (PCBN]. This TMA contains 70 visceral metastasis tissues from liver, lungs, lymph node and kidney as well as 51 bone metastatic cores from 45 metastatic CRPC (mCRPC) cases following rapid autopsy procedure. Of these, 13 patients showed t-NEPC transformation and the rest were classified as mCRPC adenocarcinoma. Our results indicate that 10 out of 13 t-NEPC patients stained positive for Pax5 expression (Fig. 4E). Again, Pax5 expression is not detected in metastatic CRPC (mCRPC) cases diagnosed with adenocarcinoma, but were present in those with t-NEPC, which supports that Pax5 expression is specific to the NE-like lineage differentiation of PCa.

Earlier reports indicate that Pax5 is one of the most important transcription factors for AR-independent cell growth [12], further supporting our findings. Pax5 is also expressed by infiltrating leukocytes surrounding the tumor [65]. To confirm that high Pax5 expression in tumor tissue actually originates from the epithelial cells/NE-like cancer cells, we further analyzed single-cell RNA-sequencing data published recently (GSE137829) [21]. Our analysis validated that adenocarcinoma does not usually express Pax5, and cancer epithelial cells start expressing Pax5 only during NE-like transformation (Fig. S4C**)**. Overall, our results validate that Pax5 is preferentially expressed during the AR-independent NE-like cancer progression.

### Pax5 is involved in the gene expression profile associated with neuronal pathways

**Fig. 5:**
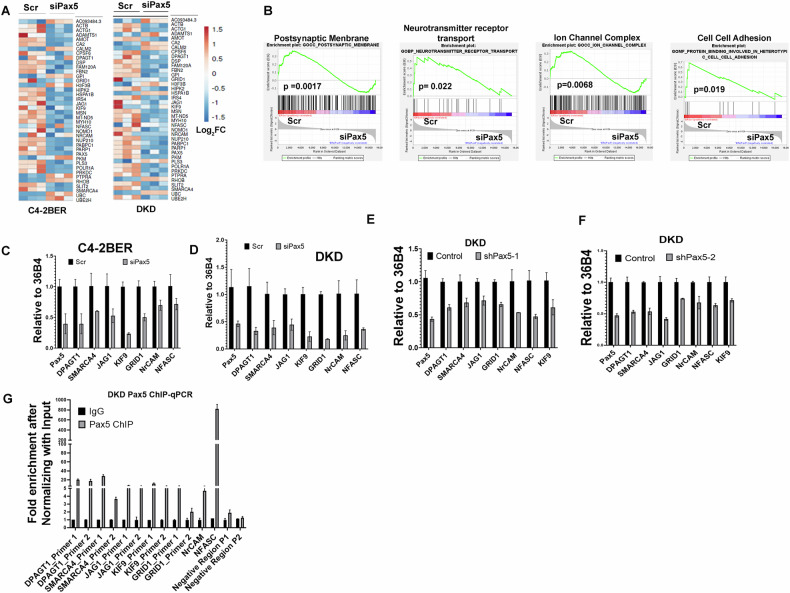
Pax5 regulates various neuronal associated pathways in NE-like cells. **A** Heatmap of Pax5-regulated gene-expression profiles for important neuronal pathways in C4-2BER and DKD upon Pax5 depletion. **B** GSEA pathway analysis using differentially regulated Pax5 genes in NE-like cells upon Pax5 depletion. **C**, **D** Validation of Pax5-regulated gene-sets in NE-like cells C4-2BER and DKD) following transient depletion of Pax5 by siRNA. Error bars represent standard errors between *N* = 3 biological replicates. **E**, **F** Validation of Pax5-regulated gene-sets in NE-like cells (DKD) following transient depletion of Pax5 by doxycycline-inducible two independent shRNAs. Error bars represent standard errors between *N* = 3 biological replicates. **G** ChIP-qPCR showing enrichment of Pax5-regulated genes over negative controls PGM5 (using two primer sets P1 and P2) and IgG.

To identify Pax5-related genes in NE-like cancer, we performed RNA-Seq under Pax5 depletion in C4-2BER and DKD cells. The resultant heat map indicated significantly differential genes between control and Pax5-depleted condition in both t-NEPC cell lines (Fig. 5A). GSEA pathway analysis of significant differentially regulated genes (from Fig. 5A) revealed that Pax5-regulated genes are involved in neuronal synapses and neuronal adhesion signatures in NE-like cells (Fig. 5B). Using qPCR in DKD and C4-2BER t-NEPC cell lines, we validated those various genes, such as NFASC, NrCAM, GRID1, SMARCA, etc., involved in neuronal pathways from Fig. 5B are downregulated upon depleting Pax5 expression (Fig. 5C–F). Further, to determine the occupancy of Pax5 within the promoters of these genes, we performed ChIP-qPCR, which revealed that Pax5 binds to the regulatory regions of those genes (Figs. 5G and S5A). Similar increase of gene expression has been reported in t-NEPC patients as compared to adenocarcinoma patients [1, 66]. Interestingly, ectopic expression of Pax5 alone in adenocarcinoma cells did not transduce these cells into NE-like phenotype (Figs. S5B and S5C). We reasoned that t-NEPC transformation from adenocarcinoma, under RB1/TP53 functional inactivation background, undergoes chromatin alterations to allow Pax5 binding to neuronal gene promoters. Thus, ectopic expression of Pax5 alone without any chromatin modification is not sufficient to induce NE-like transformation. Further, we depleted Pax5 from DKD cells to analyze SYP expression as a determinant of NE-like cell behavior. Our result indicates that depletion of Pax5 does not alter the SYP expression (Fig. S5D), indicating depletion of Pax5 will not revert the neuroendocrine transformation. Overall, our results suggest that transcriptional accessibility enables Pax5 to upregulate neuronal adhesion signatures in t-NEPC.

### Depletion of Pax5 increases therapeutic efficacy of docetaxel in t-NEPC

While growing in 2D culture, NE-like cells present various neurite-like branching (Fig. S6A). These neurite-like branching are the expansion of plasma membrane, which are important for maintaining cellular communication, synaptogenesis, secretion, migration and processing cell-cell interaction networks [67–69]. During neuroendocrine differentiation, prostate cancer cells adapt neuronal characteristics and show neurite-like protrusion [67, 70, 71]. In neurons, neurite processes are involved in trans-synaptic communication (secretion of synaptic vesicles and uptake), and therefore plays a vital role in healthy neuronal activity [72]. Like synaptic communication, t-NEPC cells upregulates their secretory function for maintaining trans-cellular communication [19, 73, 74]. Earlier, we showed that by maintaining secretory function, NE-like cells communicate with each other to enhance their therapy-resistance characteristics [19]. Whether this stabilization of neurite structure is important for resisting therapeutic stress in t-NEPC, remains elusive. Our RNA-seq data also indicated that Pax5 downstream signaling is important for synaptic communication (Fig. 5B). We found that depleting Pax5 in NE-like C4-2BER cells reduced the formation of such neurite-like branching (Fig. S6A), thereby suggesting that Pax5 is involved in neurite-like morphogenic processes in t-NEPC. In neurons, several reports indicate that these neurite processes require NCAM1 (CD56) adhesion proteins to stabilize synaptic communications [75, 76].

**Fig. 6:**
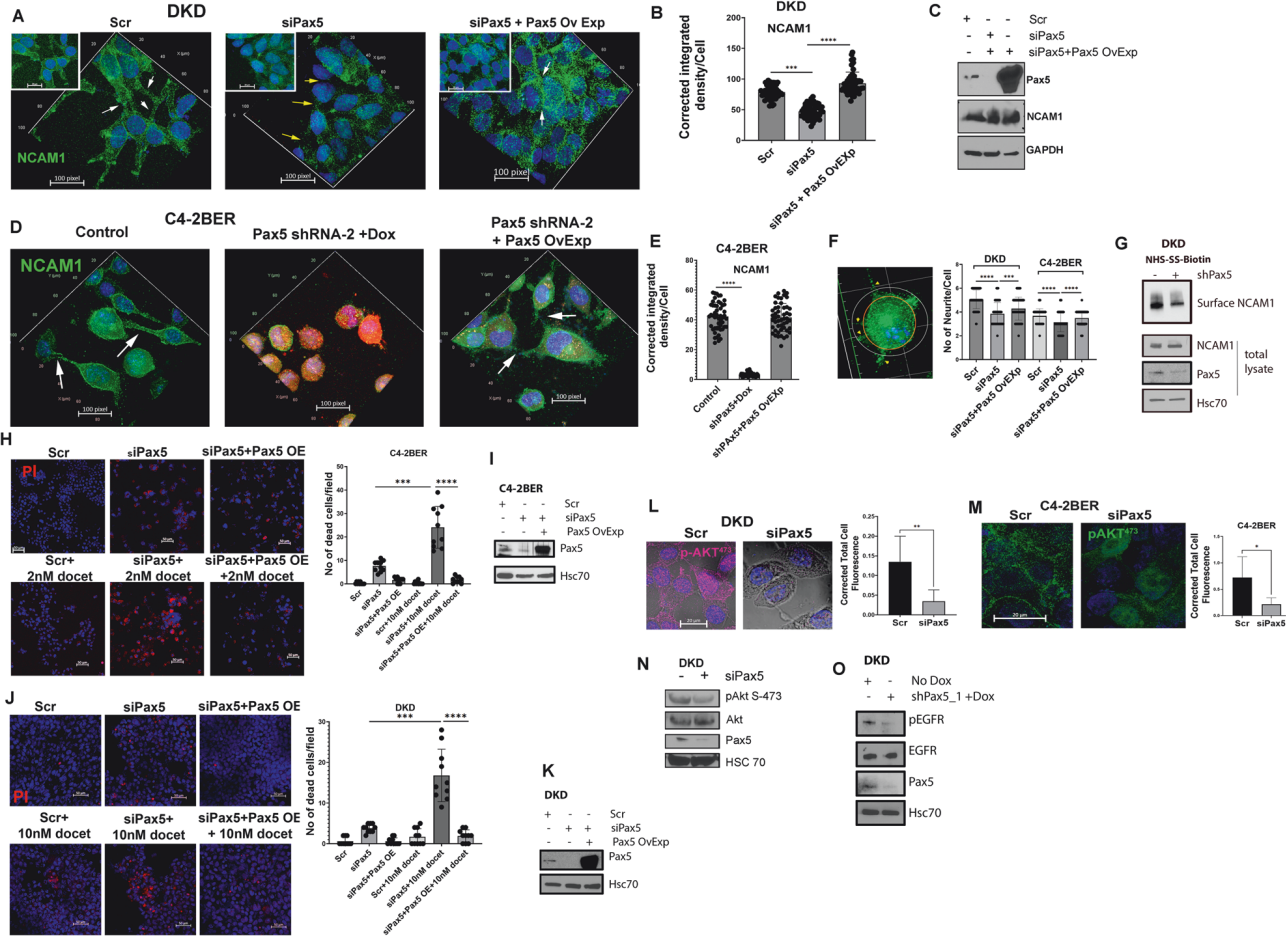
Depletion of Pax5 affects the cellular communication and increases therapeutic efficacy. **A**–**E** Immunofluorescence for surface expression of NCAM1 in DKD and C4-2BER cells following Pax5 depletion either by siRNA or by shRNA (doxycycline inducible where shRNA expression is indicated by red fluorescence) or ectopic expression of Pax5 in Pax5 knockdown cells. For C4-2BER, control was transfected with shPax5 but doxycycline was not added. Arrows indicate the NCAM1 positive neurite-like protrusion in close proximity of another cells. (White arrows showing NCAM1 positive neurite-like structure in close proximity of neighboring cells whereas yellow arrows showing loss of NCAM1 positive neurite). Inset represents the total cellular field in **A**. Image was captured in Z-stacks. 3D reconstitution was made in Zeiss Zen blue software. **A**, **D** represent the 3D reconstitution image. Super plots representing the quantification of surface localization of NCAM1 for **A** and **D** respectively. ***P < 0.0001, **P < 0.001, and *P < 0.01. Error bars represent standard errors between biological replicates of Pax5 knockdown sample. **C** Immunoblot showing Pax5 and NCAM1 expression under depletion and ectopic expression of Pax5 in DKD. DAPI represents nucleus. Scale Bar is 100pixel. **F** Super plot represents the quantitation of neurite number in C4-2BER and DKD under Pax5 depletion and Pax5 overexpression respectively. **P < 0.001 calculated through Student’s *t* test. Cell body is marked with orange circle. From orange circle any processes touches or cross the white circle was consider neurite-like structure as shown in the images. **G** Immunoblot showing NCAM1 expression in biotinylated pull-down sample under control and Pax5-depleted condition in DKD. Total protein has also been shown under control and Pax5 depletion. **H**–**K** Cell viability under chemotherapeutic stress was quantified by Propidium iodide staining in DKD and C4-2BER cells respectively under the presence and absence of Pax5. Images in **H**, **J** represent the cell death analysis. Recovery experiments was carried out by ectopically expressing Pax5 under Pax5 knockdown condition. Hoechst (blue) represent the nuclear staining. Docetaxel was added at a 5 and 10 nM concentration in C4-2BER and DKD (as per their IC50 value) respectively. ****P < 0.0001 calculated from t-Test. Error bars represent standard errors of cell death calculated from (*N* = 3) biological triplicates. Image **I** and **K** represent the knockdown and overexpression of Pax5 in DKD and C4-2BER. **L**, **M** Immunofluorescence image showing phospho-AKT S-473 surface staining (magenta for DKD and green for C4-2BER) under presence and absence of Pax5 in DKD and C4-2BER cells; bar graph showing quantification. *P* < 0.01 is * calculated from t-Test. DAPI represent the nucleus staining. **N** Immunoblot for phosphor-AKT 473 and total AKT in DKD under control and Pax5 knockdown condition. Knockdown efficiency was measured by analyzing Pax5 expression. Hsc70 represent the loading control. **O** Immunoblot represent the phospho-EGFR vs total EGFR expression under control and Pax5 knockdown condition in DKD. Hsc70 represent the loading control.

Interestingly, NCAM1 is solely expressed in t-NEPC but not in CRPC-adenocarcinoma cells [77, 78]. Therefore, presence of NCAM1 in the cell surface of t-NEPC might be important for maintaining neurite like processes to regulate synaptic communication (either through secretion or direct interaction with other cells), which is important for t-NEPC survival. Our results showed that depletion of Pax5 disrupts surface localization of NCAM1 (Fig. 6A–E and S6B–G). However, depletion of Pax5 did not alter total expression of NCAM1 (Fig. 6C and S6F). Interestingly, the ectopic expression of Pax5 following its depletion, rescues the cell surface distribution of NCAM1 (Figs. 6A–E and S6B–E). Furthermore, our results suggest that, with reduction of surface NCAM1 localization under Pax5 depletion, the neurite like processes are also diminished in number, which are again re-established with Pax5 overexpression (Fig. 6F). Using surface biotinylation assay followed by immunoblot, we additionally validated that depletion of Pax5 decreases the cell surface localization of NCAM1 without affecting its total protein levels (Fig. 6G). Overall, our results indicated that Pax5-mediated downstream signaling might be important for maintaining neurite-like protrusion with surface-localized NCAM1.

To understand whether, by regulating such neurite structure, Pax5 downstream is involved in the evasion of therapeutic stress in NE-like cells, we have performed a cell death assay by exposing these NE-like cells to the first-line chemotherapies. Earlier, we showed that these NE-like cells are highly resistant to the 1st line chemotherapy such as docetaxel [19]. Our results showed that Pax5 depletion sensitized these aggressive NE-cells towards docetaxel, however, ectopic expression of Pax5 under docetaxel treatment significantly rescued the cell death (Fig. 6H–K). This shows that Pax5 axis is associated with cell survival processes under chemotherapeutic stress.

To understand the mechanism, we found that depletion of Pax5 decreases AKT phosphorylation (Fig. 6L–N), which is an important intermediate to promote therapy-resistance [79–81]. AKT signaling is hyper-activated in neuroendocrine cancers [79, 82], and we found similar results in our cell lines, where we showed that neuroendocrine cells maintain a higher AKT activation as compared to adenocarcinoma cells (Fig. S6H). As AKT activation is mostly dependent on the growth factor receptor mediated signaling axis, therefore, we analyzed growth factors receptor localization in the surface. Here we hypothesized that by maintaining stable neurite-like protrusion, NE-like cells retain the surface localization of growth factor receptors, which can induce downstream AKT signaling. In neuroendocrine cancer, EGFR mediated signaling is highly activated, responsible for the disease’s adversity [83–85]. Therefore, to test our hypothesis, we have stained the NE-like cells with growth factor receptors such as EGFR. Our results showed that Pax5 depletion decreases the cell surface localization of tyrosine kinase receptor EGFR (Fig. S6I). Depleting Pax5 functions further diminishes the phosphorylation or activation of EGFR (Fig. 6O) without altering their total protein levels. Cell surface localization of these growth factor receptors allows them to interact with their ligands, thereby promoting downstream AKT signaling. This loss of growth factor receptors from the cell surface upon Pax5 depletion, decreases AKT phosphorylation and therefore diminishes the survival signature of t-NEPC cells. Altogether, Pax5 is crucial for maintaining neurite protrusions in conferring docetaxel resistance in t-NEPC.

### Pbx1 regulates Pax5 expression in NE-like prostate cancer

**Fig. 7:**
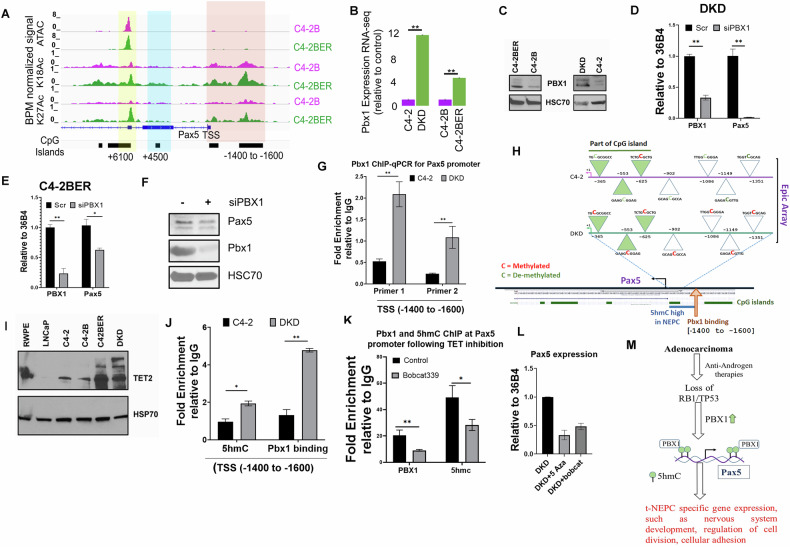
Pbx1 regulates Pax5. **A** ATAC-seq and H3K18/H3K27 acetylation ChIP-seq signal near the Pax5 promoter of C4-2B and C4-2BER cells. **B** Expression of Pbx1 in DKD vs C4-2 and in C4-2BER vs C4-2B. **p < 0.001 by Student’s *t* test. Error bars represent the standard deviation between *N* = 3 biological replicates. **C** Immunoblots for Pbx1 expression in adeno and NE-like cells. HSC70 represents the loading control. Immunoblots are representative of *N* = 3 independent experiments. **D**, **E** RT-PCR represent the Pax5 expression following the depletion of Pbx1 in the DKD and C4-2BER cells, respectively. **P < 0.001 and *P < 0.01 through Student’s *t* test. Error bars represent standard errors between *N* = 3 biological replicates. **F** Immunoblot for the Pax5 expression following Pbx1 depletion in C4-2BER cells. HSC70 is the loading control. **G** Pbx1 ChIP-qPCR was carried out using the primers designed upstream of TSS of Pax5. –ve sign represents downstream of Pax5 promoter TSS. Number represents the position near which Pax5 primers have been developed. Pbx1 binding at Pax5 promoter was compared in adeno and NE-like state. Enrichment of Pbx1 binding was calculated with respect to IgG control after normalization of inputs. **P < 0.001 and *P < 0.01 by Student’s *t* test. Error bars represent standard deviation between *N* = 3 biological replicates. **H** Schematic of Epic methylation array analysis of Pax5 gene in DKD and C4-2 cells. Green triangles represent the region that falls under the CpG island. Cytosine methylation status at the proximal promoter of both the cell lines was magnified and sequence has been shown to indicate the methylation status in each region. Red represents methylated/modified cytosine whereas green is demethylated cytosine. Pbx1 binding sight at the promoter is represented by arrow. **I** Immunoblot for TET2 expression in various prostate cancer cell lines. HSP70 functions as loading control. **J** 5hmC footprint mark was analyzed by ChIP-qPCR at Pbx1 binding site of Pax5 promoter region. Fold enrichment of 5 hmC footprint as well as Pbx1 binding was calculated with respect to IgG control after normalization of inputs. *P < 0.01 and **P < 0.001 by Student’s *t* test. Error bars represent standard deviation between biological replicates. **K** 5hmC and Pbx1 ChIP-qPCR was carried out following the treatment of DKD cells with Bobcat339 (50 μM for 18–24 h) and compared with control untreated sample. Primer 1 (Pbx1 binding site for Pax5) was used here to amplify the ChIP-enriched region. *P < 0.01 and **P < 0.001 by Student’s *t* test. Error bars represent standard deviation between *N* = 3 biological replicates. Fold enrichment of 5hmC footprint as well as Pbx1 binding was calculated with respect to IgG control after normalization of inputs. **L** RT-PCR was carried out to analyze the Pax5 gene expression following treatment of DKD cells with 5-Azacytidine (0.1 μM for 5–7 days) and Bobcat339. **M** Schematic representing the overall Pbx1/Pax5 regulation in t-NEPC maintenance.

Pax5 expression is highly selective for NE-like cancer but not for prostate adenocarcinoma. This selective expression of Pax5 urges us to investigate its transcriptional regulatory mechanism. Therefore, we next investigated into the molecular events that lead to the preferential upregulation of Pax5 in NE-like cells. During NE-like differentiation, cells become independent of the AR signaling axis. Therefore, we questioned whether depletion of AR is sufficient for preferential upregulation of Pax5 in NE-like cells or whether AR depletion is essential for additional modification, to induce Pax5 expression. Our results showed that immediate (5–7 days) inhibition of AR nuclear translocation by addition of AR-antagonist, enzalutamide, or apalutamide, did not induce Pax5 expression in the NE-like cells (Fig. S7A, B). This suggests that short-term depletion of AR activity is not sufficient for Pax5 expression. Interestingly, while studying the Pax5 promoter regions, we found enhanced histone acetylation nearby the Pax5 promoter region of NE-like cells (C4-2BER) as compared to the adenocarcinoma cell line (Fig. 7A). Additionally, our i*n vitro* ATAC-seq data validated that these loci are more accessible in NE-like cells as compared to adenocarcinoma (Fig. S7C). Overall, these data indicated that the Pax5 promoter is transcriptionally active in NE-like cells, and loss of functional AR is not an immediate driver of Pax5 expression.

To understand what drives Pax5 expression in NE-like cells, we analyzed the consensus TF-binding motifs at the transcriptionally active Pax5 promoter. Using the Biobase-transfac Gene regulation database and Transfac TF screening tool (Qiagen), we screened prospective transcription factor motifs within the Pax5 promoter (+500 to -2000bp) (Fig. S7D). Based on the scores of MATCH, we selected the top 10 transcription factors (Fig. S7E). After carefully comparing those top 10 TFs with t-NEPC patients’ gene expression retrieved from GEO and SU2C databases, we found that Pbx1 shows a consistently higher overexpression specifically in t-NEPC cohorts (Fig. S7F); however, other TFs such as MYB, Pax2 or HoxA3 are not exclusively overexpressed in all the patient databases as shown in Fig. S7F. Moreover, Pbx1 overexpression is consistent with upregulated Pax5 expression specific to t-NEPC (Fig. S7F). In line with the above findings in patient databases, Pbx1 is also upregulated in our NE-like cell lines (Fig. 7B, C). Indeed, the increased ATAC-seq signal in C4-2BER cells upstream of Pax5 overlaps with the Pbx1 motif (Fig. S7C), suggesting that activity of this Pbx1 binding may control Pax5 expression (Fig. S7F). Further, we investigated whether loss of function of AR is inducing the Pbx1 expression, we analyzed the Pbx1 expression in AR-null cell lines PC3. Our data indicate that PC3 has comparatively low expression of Pbx1 as compared to DKD (Fig. S7G). These results therefore indicate that there is no direct correlation of functional AR loss and Pbx1 mediated Pax5 expression.

To investigate whether Pbx1 regulates Pax5 expression, we depleted Pbx1 from NE-like PCa cells and observed a decrease in Pax5 expression (Fig. 7D-F). Using ChIP-qPCR, we tested whether Pbx1 binds at the differentially accessible promoter region of ATAC-seq peaks in the NE-like cells. Our result showed that Pbx1 occupancy is highly enriched upstream of the Pax5 TSS (within -1400 to -1600bp) (Fig. 7G). These results indicate that Pbx1 acts as a putative regulator of Pax5 gene expression in NE-like cells.

Although PBX1 expression increases significantly from adenocarcinoma towards t-NEPC transformation, we questioned to understand what favors differential recruitment of PBX1 in Pax5 promoter in NE-like cells but not in adenocarcinoma. Interestingly, the Pbx1 binding site (ATAATTACT) falls within two well-conserved CpG islands within the Pax5 promoter. To determine whether these CpG islands have any potential effect on differential chromatin accessibility (as shown in ATAC-seq peaks analysis from Fig. 7A) between the adenocarcinoma and NE-like PCa, we investigated into the Pax5 promoter methylation status. To determine whether demethylation of CpG region favors chromatin accessibility at the Pax5 promoter, we analyzed the promoter methylation status of adeno and NE-like cells by performing EPIC Methylation Array between adenocarcinoma (C4-2) and NE-like cells (DKD). Our results indicate that in NE-like cells, the Pax5 promoter is heavily methylated compared to adenocarcinoma cells (Fig. 7H and Table S2) and therefore raised the question of how a methylated Pax5 promoter becomes accessible for Pbx1 binding. Recent studies have shown that hydroxymethylation can alter the chromatin compactness to a state that favors gene expression [86, 87]. However, bi-sulfite treatment as performed in EPIC sequencing cannot distinguish between methylation and 5′-hydroxymethylation of cytosine (5hmC) [88]. Hydroxylation of 5-methyl cytosines is carried out by the Tet-family (Ten-Eleven Translocation) of enzymes [89]. We therefore analyzed the Tet expression in the patient database and found that Tet2 expression is often upregulated in NE-like PCa (Fig. S7H). We measured protein expression of Tet2 in both adeno and NE-like PCa cell lines by western blotting and found that TET2 expression is increased in NE-like cell lines compared to adenocarcinoma (Fig. 7I). To understand whether increased expression of TET2 correlates with hydroxymethylation at Pax5 promoter in NE-like PCa cells, we tested 5hmC level at Pax5 promoter by ChIP-qPCR using a 5hmC antibody. We found specific enrichment of 5hmC peaks at Pax5 promoter CpG islands in NE-like cells (Fig. S7I). One of these 5hmC regions overlaps with Pbx1 motif and falls precisely at Pax5 promoter (Fig. S7I, TSS -1400 to -1600bp). Interestingly, our results showed that the 5hmC footprint at the upstream Pbx1 binding site of Pax5 promoter is higher in NE-like cells than in adenocarcinoma (Fig. 7J). Overall, our results indicate that Pax5 promoter regions are differentially hydroxymethylated in NE-like cells, thereby, leading to chromatin relaxation and Pax5 expression. To validate that 5hmC can induce the Pbx1 binding at the promoter regions of the Pax5 promoter; we prevented hydroxymethylation by inhibiting Tet activity with a specific Tet inhibitor, Bobcat339. We observed that inhibition of TET activity reduced Pbx1 binding at the 5hmC sites of the Pax5 gene (Fig. 7K), and reduced Pax5 expression (Fig. 7L). Alternatively, depleting Tet2 levels in NE-like DKD cells showed a similar reduction of Pax5 expression (Fig. S7J). Additionally, demethylation of the Pax5 promoter region (by treating with 5-azacytidine for a week) also decreases Pax5 expression (Fig. 7L); thereby indicating that hydroxymethylation of cytosine is pivotal in recruiting Pbx1 to induce Pax5 expression. Together, our results showed that the hydroxymethylation of cytosine within Pax5 promoter favors Pbx1 binding to initiate the Pax5 expression in NE-like PCa cells.

Although, ectopic expression of Pbx1 did not induce NE-like transition or morphology to the adenocarcinoma cells (Fig. S8A, B), however, its depletion from NE-like cancer affects neurite formation similar to Pax5 deletion (Fig. S8C, D). These results suggest that, similar to Pax5, Pbx1 alone cannot induce NE-like signature but is important for maintaining the neurite projections through regulating Pax5 expression.

Overall, our results suggest that Pax5 in t-NEPC is pivotal to maintaining neurite processes with surface-localized NCAM1, thereby activating downstream survival signals. Perturbation of such Pax5-mediated functions renders the NE-like cells more sensitive to docetaxel chemotherapies (Fig. 7M).

## Discussion

The use of new-generation anti-AR therapies may enhance the overall survival of CRPC cases; however, the resistant clones are highly aggressive and present in various clonal forms. Among these resistant populations, nearly 20% showed NE-like transformation with a distinct genetic signature compared to the adenocarcinoma sub-type [17]. NE-like cancers are highly lethal and frequently responsible for the over-spread of the cancers, which is difficult to manage. Although pathologically, these cancers are highly heterogeneous, they do follow some common genetic signatures [90, 91]. Moreover, while the origin of therapy-induced trans-differentiation is still under investigation, recent reports indicate that, genetic signature of these cancers maintains distinct similarities with de novo neuroendocrine cancers, including small-cell lung cancers [57]. This suggests that a common mechanism underlines the clinical manifestation of NE-like PCa. In this study, by comparing chromatin accessibility, epigenetic signatures, and gene expression between adeno and NE-like cancers, we have identified Pax5 as a transcription factor important during NE-like transformation.

While analyzing the nature of gene expression associated with NE-like trans-differentiation processes, our results indicated that pathways associated with neuronal differentiation, axonogenesis, neuronal communications are selectively enriched in NE-like cancers, as reported elsewhere [12, 91, 92]. Our results demonstrate that increased expression of neuronal genes in NE-like cancers is associated with increased marks of transcriptionally active chromatin, including accessibility and histone modifications. While we focus on the chromatin activity status of promoters, it is likely that altered chromatin status at enhancer regions also contribute to t-NEPC transformation, and future work using Hi-C and HiChIP to understand enhancer-promoter interactions will likely reveal new insights in t-NEPC transformation. Currently, our results indicate that many differential genes have altered promoter chromatin status at Pax5 binding sites. Pax5 expression has been validated in various NE-cohorts, including PDX models. Further studies with metastatic TMA revealed that some of the neuroendocrine patients are negative for Pax5 expression; indicating either stage-specific or clone-specific expression of Pax5 associated with NE-like trans-differentiation. Similar to CRPC, Pax5 expression has been detected in neuroendocrine lung cancer as well as N-type neuroblastoma cells and reported to be associated with aggressive nature of cancers [93, 94]. Interestingly, in neuroendocrine lung cancer, heterogeneous expression of Pax5 has been reported with the highest in the small-cell (SCLC) subtype, followed by large cell and carcinoid [93]. With the genetic similarities of SCLC and NE-like PCa [61, 62], our results suggested similar heterogeneity in patient sample and thereby warrants more in-depth investigation with a larger cohort. The absence of Pax5 in any of the adenocarcinoma tissue indicates that Pax5 expression might be specific transcriptional event in NE-subtype. However, IHC from metastatic TMA revealed that one core of LuCaP173.2A, which is represented as double negative prostate cancer (DNPC; AR negative with no expression of classical NE markers such as CHGA and SYP but express EZH2 and MYCN), also expresses a low level of Pax5. The report indicates that this LuCaP173.2 with the serial passage starts expressing NE markers like SYP; therefore, specifying that such NE-differentiation might be a disease continuum from the double negative PCa [1]. Therefore, our study warrants further investigation with a larger cohort of double negative patient tissue to justify the Pax5 expression during differentiation processes. Recently, association of Pax5 has been predicted in AR-independent growth of CRPC [12]. Moreover, another Pax group of molecules, known as Pax6, has been shown with prostate NE-like transformation [57]. Overall studies suggest the importance of Pax-group of transcriptional regulators in the development of NE-like cancers.

While studying the underlying mechanism of Pax5 expression in NE-like cancer, we observed the involvement of Pbx1 in Pax5 transcription. Our results indicate that Pbx1 is selectively overexpressed in NE-like cancers, which was further validated in patient’s gene expression data. Pbx1 expression has also been observed in xeno-transplanted neuroendocrine prostate cancer [95]. Despite these connections, the functional importance of Pbx1 has not been investigated in t-NEPC. Pbx1 has two isoforms. Although Pbx1 expression is observed in adenocarcinoma, whether isoform specific expression of Pbx1 has any role in t-NEPC needs further investigation. Our results presented here reveal the crucial importance of Pbx1 in NE-like PCa. Our ATAC-seq data shows NE-specific chromatin accessibility at the Pbx1 binding site within the Pax5 promoter, along with hydroxymethylation of cytosine in CpG islands. It has been shown that high-5hmC is an adverse predictor for biochemical recurrence of ERG-negative prostate cancers [96] and can function as a prognostic marker for PCa development [97]. Our study highlights the importance of examining 5hmC relative to gene expression in NE-like transformation. While we demonstrate 5hmC at the Pax5 promoter, future studies using genome-wide methods of measuring 5hmC will reveal the overlap between gene activation, chromatin accessibility, histone modification, and promoter 5hmC signatures in specific subtypes of NE-like trans-differentiation.

Our results indicate that Pax5 is involved in the transcription of specific neuronal gene signatures in NE-like cancers. As neuronal axis is one of the major axes for the prostate cancer growth and survival (following AR axis) [54, 98], inhibiting such axis can sensitize cancer cells to the therapy. This is especially important for NE-like cancer, which is highly dependent on the neuronal axis for its survival [99]. In these regards, the identification of Pax5 as an important regulator for neuronal axis of PCa is highly significant. Depletion of Pax5 not only decreases neurite-like protrusion but also induces cells to respond to the docetaxel therapies. This decrease in neurite projection can disrupt trans-cellular communication of NE-like cells, thereby sensitizing them to therapeutic stress [19]. In this context, our data indicates that Pax5-mediated stabilization of NCAM1 in the cell surface is significant for its docetaxel-resistance. Although, NCAM1 expression, along with SYP and CHGA are the predictive markers for neuroendocrine transformation [100], however, NCAM1 may not be a merely marker for t-NEPC, rather localization of NCAM1 can be important for studying therapy-resistance in t-NEPC. Although, how Pax5 depletion alter the surface localization of NCAM1 need to be studied in depth. Targeting NE-like PCa is challenging; therefore, identification of Pbx1/Pax5-regulated function is beneficial for the development of future therapeutic strategies.

Overall, our results illustrate the functional importance of PBX1/Pax5 transcriptional axis in maintaining the NE-trans-differentiation process. This is significant in terms of understanding the disease etiology as well as screening or detection of neuroendocrine transformation. Understanding such heterogeneity has broad implications, especially in developing selective therapeutic strategies. In summary, our study provides a new avenue for screening NE-like prostate cancer.

### Supplementary information

Supplementary figure table and legends

Check list

Raw blot

## Data Availability

RNA-seq, Chip-Seq and ATAC-seq data was deposited in GEO database with accession number GSE210848.
