# Supplementary material for: Understanding the function of Pax5 in development of docetaxel-resistant neuroendocrine-like prostate cancers
Source: Cell Death Dis. 2024 Aug 25;15(8):617. doi: 10.1038/s41419-024-06916-y (PMC11345443; doi:10.1038/s41419-024-06916-y)
Supplement: Supplementary file 1 — Supplementary figure table and legends [file 41419_2024_6916_MOESM1_ESM.pdf]

Supplementary Table 1: List of transcription factors binding sites in and around the accessible chromatin regions.

Supplementary Table 2: Comparative analysis of EPIC methylation array between C4-2 and DKD.

Supplementary Figure legends:

Supplementary Fig. S1: AR activity in NE-like cells and Pathway analysis by GSEA, GO and IPA in t-NEPC patients and NE-cell lines: A. GSEA analysis of combined differentially regulated genes between t-NEPC and Adenocarcinoma using GSE126078, GSE66187, SU2C 2019 database. **B.** Immunofluorescence images of NeuN in C4-2, C4-2B C4-2BER and DKD cells. Bar graph showing quantitation of nuclear NeuN intensities. P<0.0001 is represented as **** by student t-test. **C.** Western blot showing AR expression in C4-2, C4-2B, C4-2BER, DKD cell lines. **D.** RT-PCR showing expression of AR-regulated KLK2 and TMPRSS2 genes in C4-2, C4-2B, C4-2BER, DKD and NCI-H660 cell lines. P<0.0001 is represented as **** by student t-test. **E-G.** GO terms (Biological processes, Pfam domains, Molecular functions) enriched in C4-2BER vs C4-2B. **H.** Ingenuity Pathway Analysis (IPA) showing variety of pathways associated with differentially expressed genes in C4-2BER. **I.** Image showing crowded morphology of NE-like DKD vs adenocarcinoma LNCaP and C4-2B in 3D suspension culture. Bar graph showing quantification of cluster length of LNCaP, C4-2B and DKD suspension cellular aggregates. P<0.001 is represented as *** by student t-test.

Supplementary Fig. S2: Chromatin accessibility correlating to gene expression in t-NEPC: A. RNA-seq data showing expression of Hox A genes in C4-2B and C4-2BER. Error bars represent standard deviation between biological replicates. All differences are statistically significant, t-test p< .05. **B**. ATAC-seq signal (BPM normalized) shown for NCAM1, VCAN and CD40 genes in C4-2B and C4-2BER. **C**. RNA-seq data showing expression of cell adhesion genes in C4-2B and C4-2BER. Error bars represent standard deviation between biological replicates. All differences are statistically significant, t-test p< .05. **D.** WB showing histone acetylation levels for H3K9, H3K18 and H3K27 in C4-2 and DKD respectively. Quantification of bands are shown in bar graph. **E.** ATAC-seq signal showing accessibility for CHGA and ASCL1 genes.

Supplementary Fig. S3: Transcription factor expression in t-NEPC patients: A. Expression of top 10 transcription factors in adenocarcinoma and t-NEPC patient cohorts from GSE 126078, GSE 66187 and SU2C_2019 respectively. **B.** Plots represent individual patient gene expression (From GSE126078) between CRPC adenocarcinoma and t-NEPC for Pax5, ETV5 and KLF12. P<0.001, P<0.0001 and P<0.00001 are represented as **, *** and **** and calculated from t-Test. **C.** Plot showing Ct values of various RT-PCR normalization controls under different condition in DKD and C4-2BER. **D.** RT-PCR for ETV5 and KLF12 in C4-2BER and C4-2BAR using GAPDH as a reference gene. **E.** ATAC-Seq and ChIP-Seq Peaks comparison between adenocarcinoma and NE-like cell line C4-2BER for TNC and DAB1 genes. Sequence below indicates the Pax5 motif within those differentially accessible gene regions. **F.** Gene expression profile of TNC and DAB1 genes between C4-2B and C4-2BER RNA-Seq data.

Supplementary Fig. S4: Validation of Pax5 expression: Immunoblot showing Pax5 expression in **A.** C4-2 vs DKD, C4-2B vs C4-2BER and DKD vs PC3 (AR null adenocarcinoma cell line). **B.** Expression of Pax5 at primary and metastatic sites of transgenic mice developed following prostate specific knockout of Pten and RB1 (GSE90891). **C.** From scRNA-seq, expression of Pax5 was analyzed in various cell population within the cancer tissues of various patients. The Red circles indicate neuroendocrine patients and Blue circle indicates adenocarcinoma patients.

Supplementary Fig. S5: Overexpression of Pax5 did not induce NE-differentiation: A. ChIP-qPCR showing enrichment of Pax5 regulated genes NrCAM and NFASC over negative controls PGM5 (using two primer sets P1 and P2) and IgG. **B.** RT-PCR for analysis of CHGA, SYP and Pax5 expression in adenocarcinoma C4-2 and C4-2B following Pax5 overexpression. DKD (neuroendocrine cell line) is used as a positive control for Pax5 expression. **C.** Immunoblot for analysis of NE-marker SYP following the ectopic expression of Pax5 in adenocarcinoma C4-2 cells. DKD is used as a positive control for Pax5 expression. HSC70 represents the loading control for immunoblot. **D.** Immunoblot for analysis of NE-marker SYP following depletion of Pax5 from DKD cells. HSC70 represents the loading control for immunoblot.

Supplementary Fig. S6: Pax5 depletion disrupts cellular interaction and improves therapy:

A. Representative Image of neurite branching of C4-2BER cells following Pax5 knockdown. Arrows indicate the neurite branching. RT-PCR to analyze knockdown efficiency of Pax5 in C4-2BER cells following depletion of Pax5. P<0.001 is ** from t-Test. Error bars represent standard errors between biological replicates (N=3) of Pax5 knockdown sample. **B-E.** Immunofluorescence images showing NCAM1 staining (green) following the depletion of Pax5 in C4-2BER and DKD cells either by siRNA or two independent shRNAs. Arrows indicate the NCAM1 positive cellular neurite like structure. White arrow showing multiple NCAM1 positive neurites in close proximity from neighboring cells; yellow arrow showing reduced NCAM1 positive neurite. Inset represents the total cellular field. Super plots showing quantification of NCAM1 surface expression for figures B and E respectively. Pax5 nuclear staining under various condition is represented in **C** and **D**. Doxycycline induction induces tRFP (Red) expression showing in the inset of image D. Bar graph represent the quantification of staining intensity. DAPI represent the nuclear staining. P<0.0001 is ***, P<0.001 is ** and P<0.01 is *. Error bars represent standard errors between biological replicates (N=3) of Pax5 knockdown sample. **F**. Immunoblot represents the Pax5 and

NCAM1 expression following Pax5 depletion by shRNA in a doxycycline inducible manner and ectopic Pax5 expression. GAPDH is the loading control. **G.** Figure represent the NCAM1 distribution under various control condition. Left indicate the NCAM1 expression (green) under doxycycline inducible non-silencing shRNA control (Inset represents the non-silencing shRNA expression in Red under doxycycline induction). Right image represents the NCAM1 expression (green) under Pax5 shRNA transfection condition without addition of doxycycline (without induction of Pax5 knockdown). Superplot represent the quantitation of surface NCAM1 expression under these conditions. **H** Immunoblot showing phospho-AKT S473 and total AKT expression in NE-like DKD vs adenocarcinoma C4-2 cells. HSC 70 is the loading control. **I.** Immunofluorescence images of EGFR (Green) in control and Pax5 depleted DKD cells. DAPI represents the nucleus. Scale Bar 20um. Inset images show the respective Pax5 depleted cell (red) and surface EGFR expression (Green). $P<0.01$ is * calculated from t-Test. Error bars represent standard errors between (N=3) biological replicates.

Supplementary Fig. S7: Validation of Pbx1 binding in Pax5 promoter: A. Pax5 expression following 10uM apalutamide treatment in various passages (over ~40 days) of C4-2B cells (represented as C4-2BAR). Initially, the growth of the cells was very slow. After 3rd passage ~30days cells were highly proliferative. Along with Pax5, NE markers like CHGA and SYP expression were also evaluated in late passage cells. Error bars represent standard errors between N=3 biological replicates. $P<0.01$ is *, $P<0.001$ is ** and $P<0.0001$ is *** by Student t-test. **B.** Immunoblot for nuclear expression of Pax5 expression under short-term treatment of enzalutamide (6 days) alone or in association with 5-Azacytidine. DKD cells were run as a positive control for Pax5 expression. Enzalutamide treatment was validated with expression of AR. 5-azacytidine treatment was validated by analyzing the expression of DNMT1. **C.** Representative ATAC-Seq

peak at Pax5 promoter region between C4-2B and C4-2BER cells. Pbx1 binding site was determined at the highlighted regions as indicated by arrow. **D.** Representation of prospective motif/transcription factor search using Biobase Gene regulation database (Qiagen) at Pax5 promoter region. **E.** Prospective top ten transcription factors that may have binding sites at the Pax5 promoter regions. **F.** Expression of top 10 prospective transcription factors at Pax5 promoter regions in different neuroendocrine and adenocarcinoma patient cohorts from GEO datasets (GSE126078, SU2C, Beltran 2016). Their expression is also compared with the RNA expression of Pax5 of respective cohorts. In addition, scatter plot for the GSE127098 for Pax5 and Pbx1 comparison is shown in right. **G.** Immunoblot for Pbx1 under NE-like DKD cells and AR null adenocarcinoma PC3 cells. Hsc70 represent the loading control. **H.** Comparison of TET protein expression in various patient cohort between adenocarcinoma and t-NEPC. **I.** 5hmC ChIP-qPCR was carried out using the primers at promoter CpG sites as well as with Pbx1 binding sites (using primer 1 sequence). Along with 5hmC footprint analysis, Pbx1 binding efficiency at those two sites was also compared following the Pbx1 ChIP. Enrichment of 5hmC or Pbx1 binding was calculated with respect to IgG control after normalization of inputs. Error bars represent standard deviation between N=3 biological replicates. **J.** RT-PCR showing Pax5 expression under TET2 depletion via siRNA in DKD cells. Error bars represent standard errors between N=3 biological replicates. $P<0.01$ is *, $P<0.001$ is ** and $P<0.0001$ is *** by Student t-test.

Supplementary Fig. S8: Pbx1 function as an upstream of Pax5 mediated processes: A. RT-PCR for analysis of neuroendocrine markers such as CHGA, SYP and Sox2 in adenocarcinoma cells following Pbx1 overexpression. The expression of these markers was compared with NE-like cell line DKD. **B.** Phase contrast microscopic images of neurite formation morphology following Pbx1 overexpression in C4-2 cells and compared the morphology with DKD cells. **C.** Phase

contrast microscopic images of neurite structure following Pbx1 depletion from NE-like DKD cells. Plot represents the quantitation of neurite structures. P<0.0001 is *** calculated from t-Test. Experiments has been repeated three times. D. Immunoblot for the expression of NCAM1 under control and Pax5 depleted condition in DKD and C4-2BER cells. Hsc70 represent the loading control.

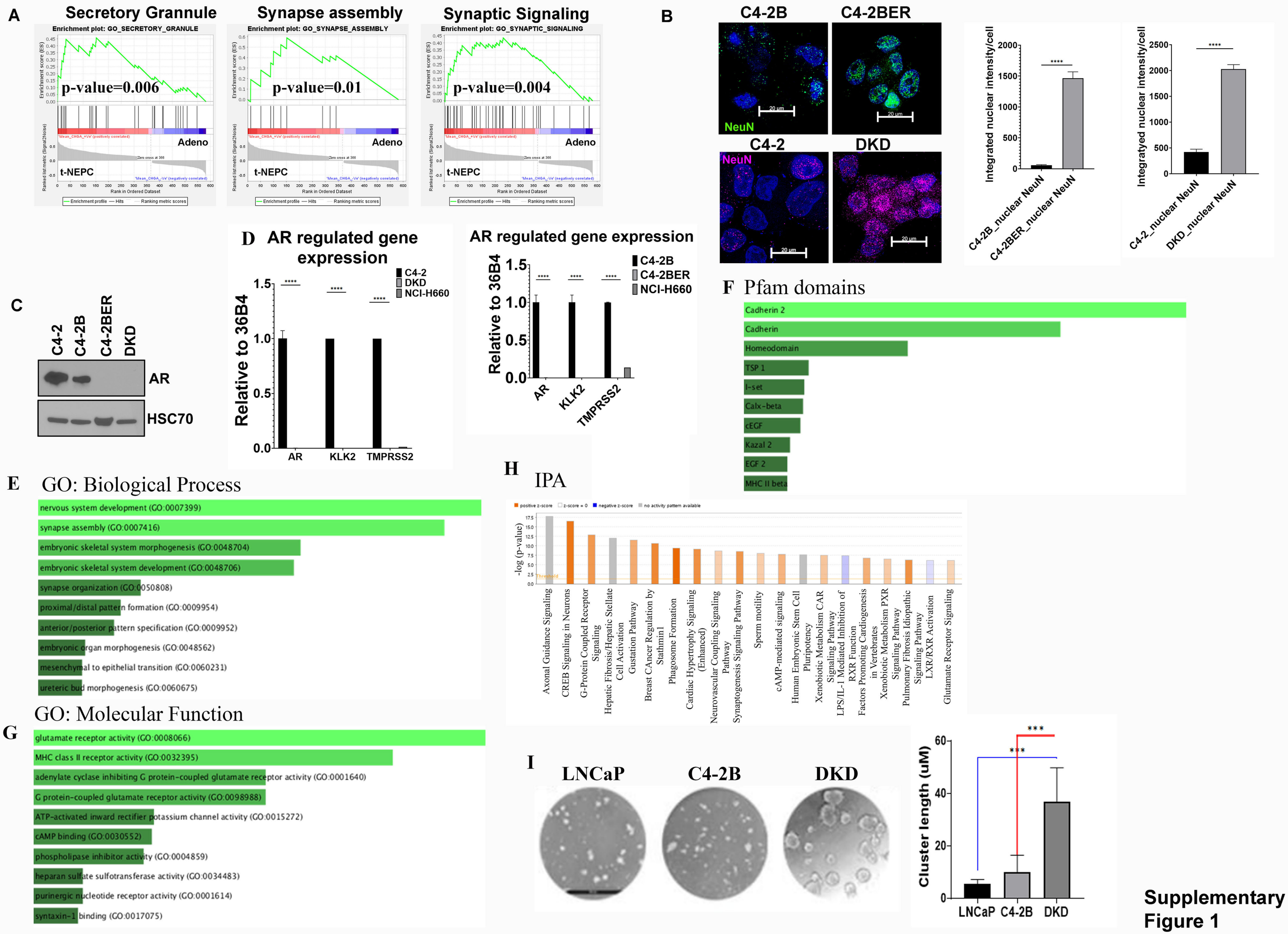

Supplementary Figure 1

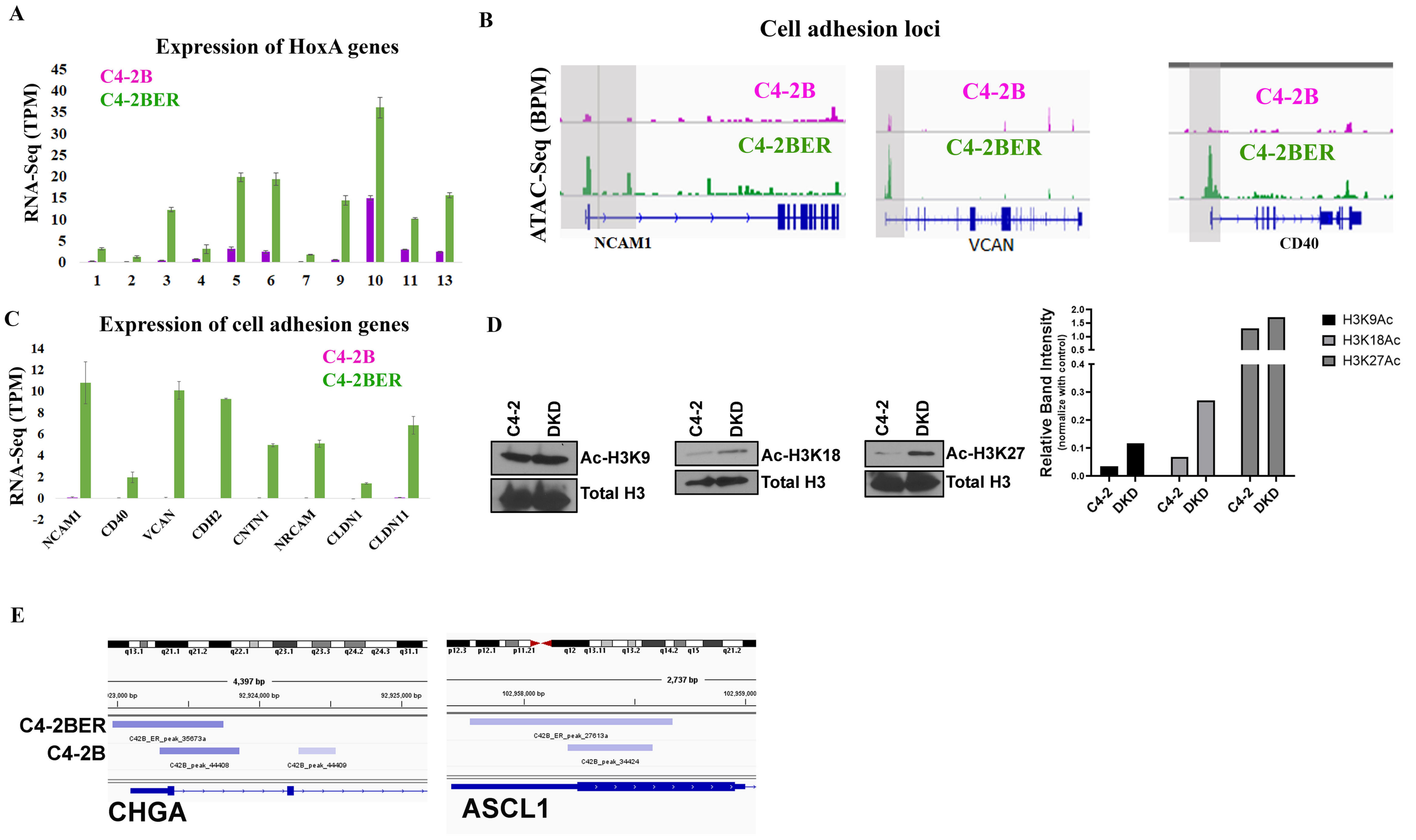

Supplementary Figure 2

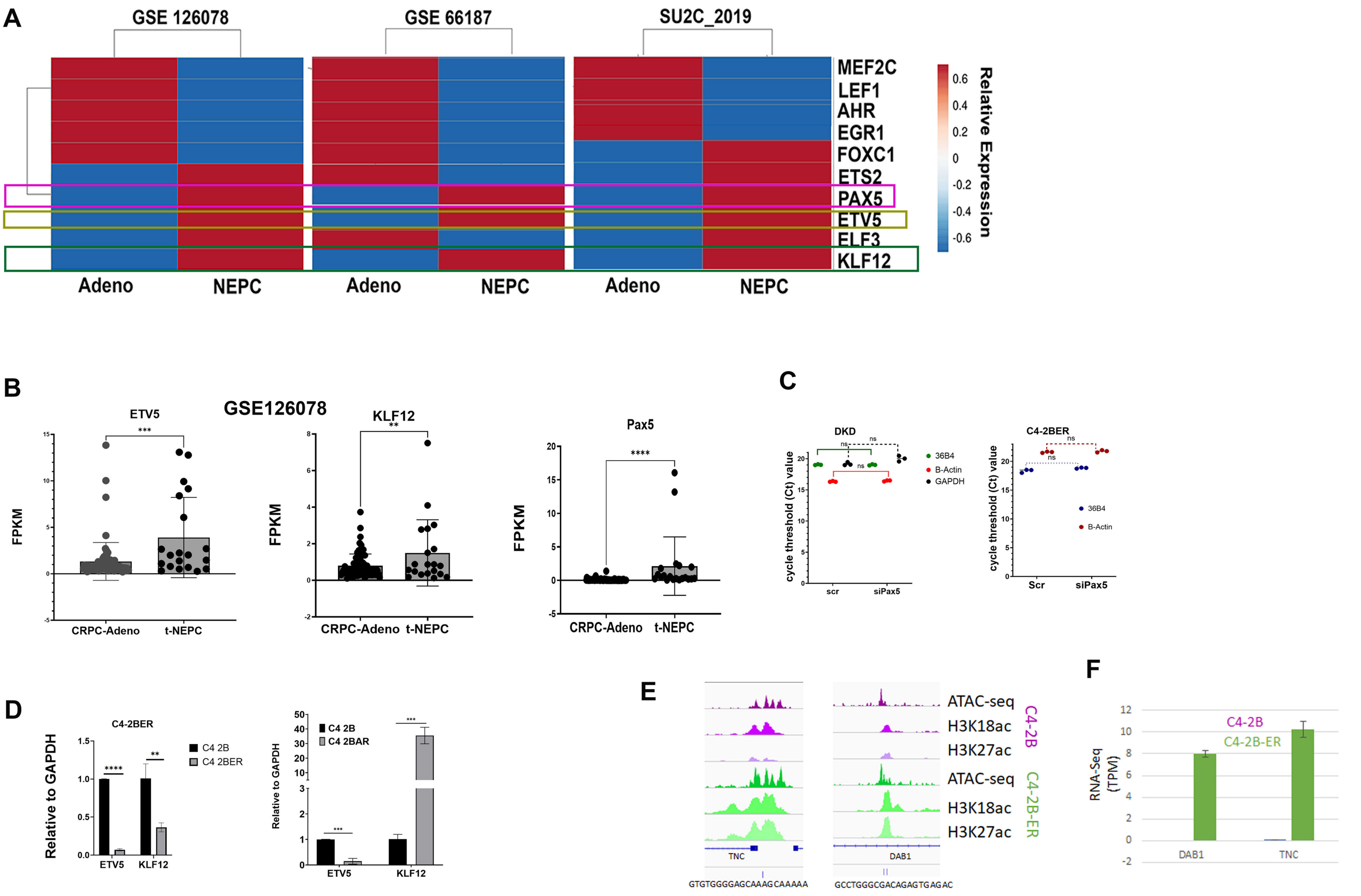

Supplementary Figure 3

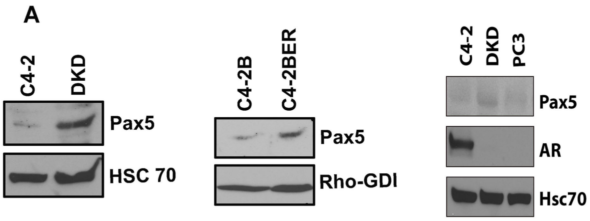

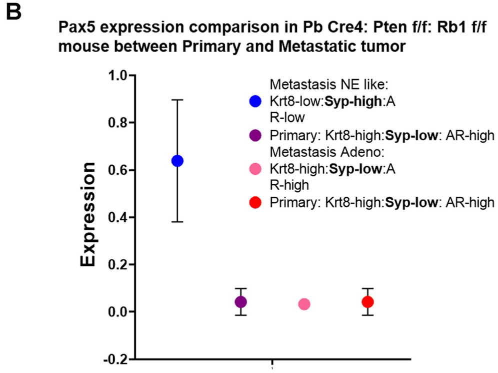

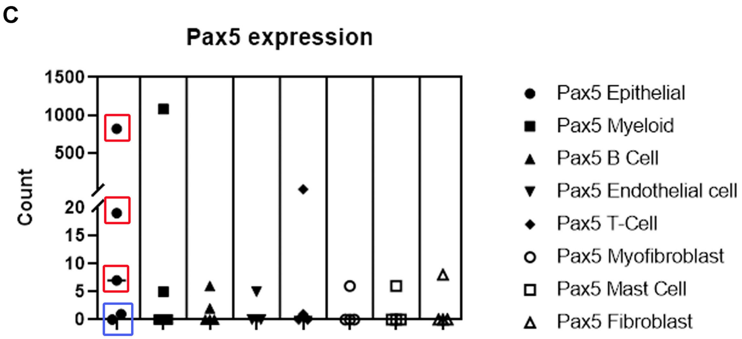

Supplementary Figure 4

A

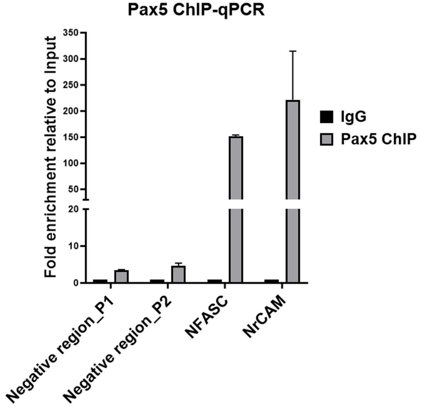

B

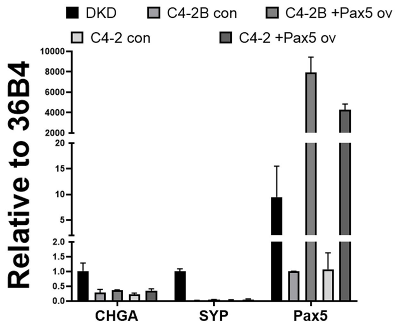

C

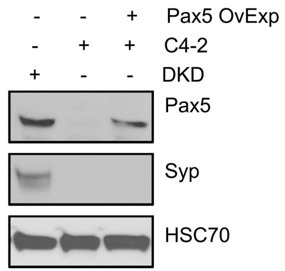

D

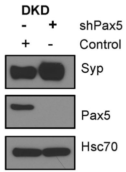

Supplementary Figure 5

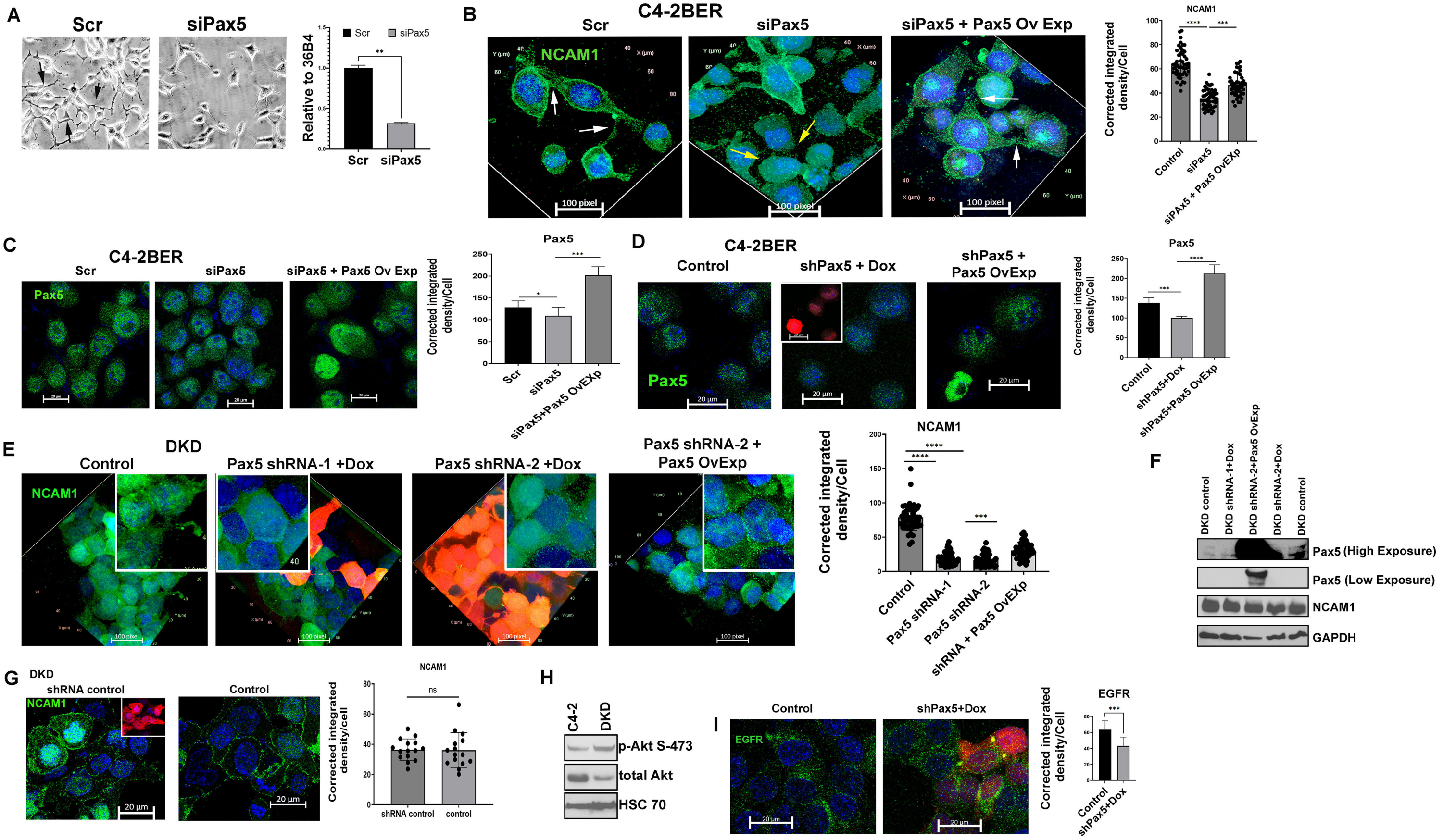

Supplementary Figure 6

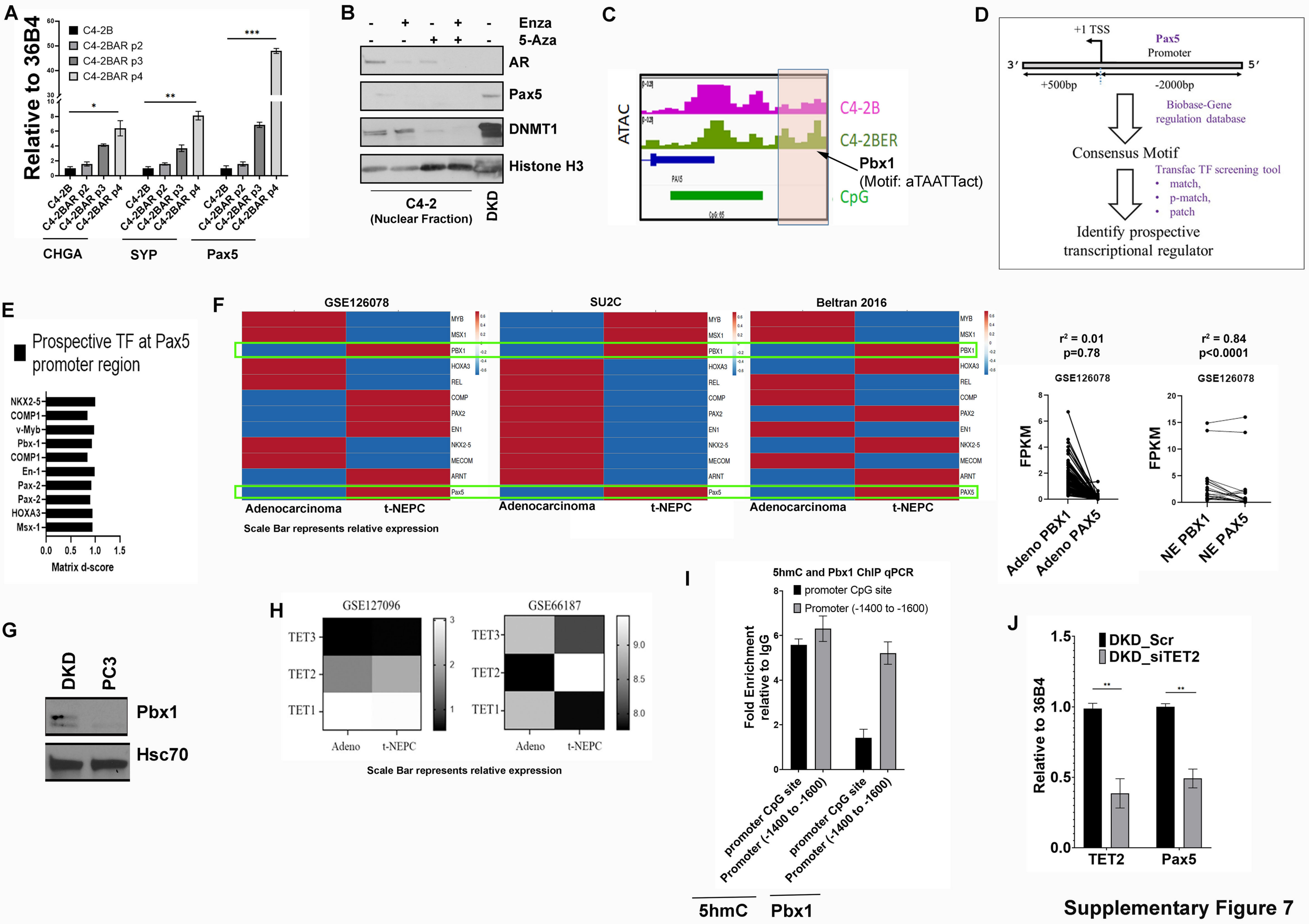

Supplementary Figure 7

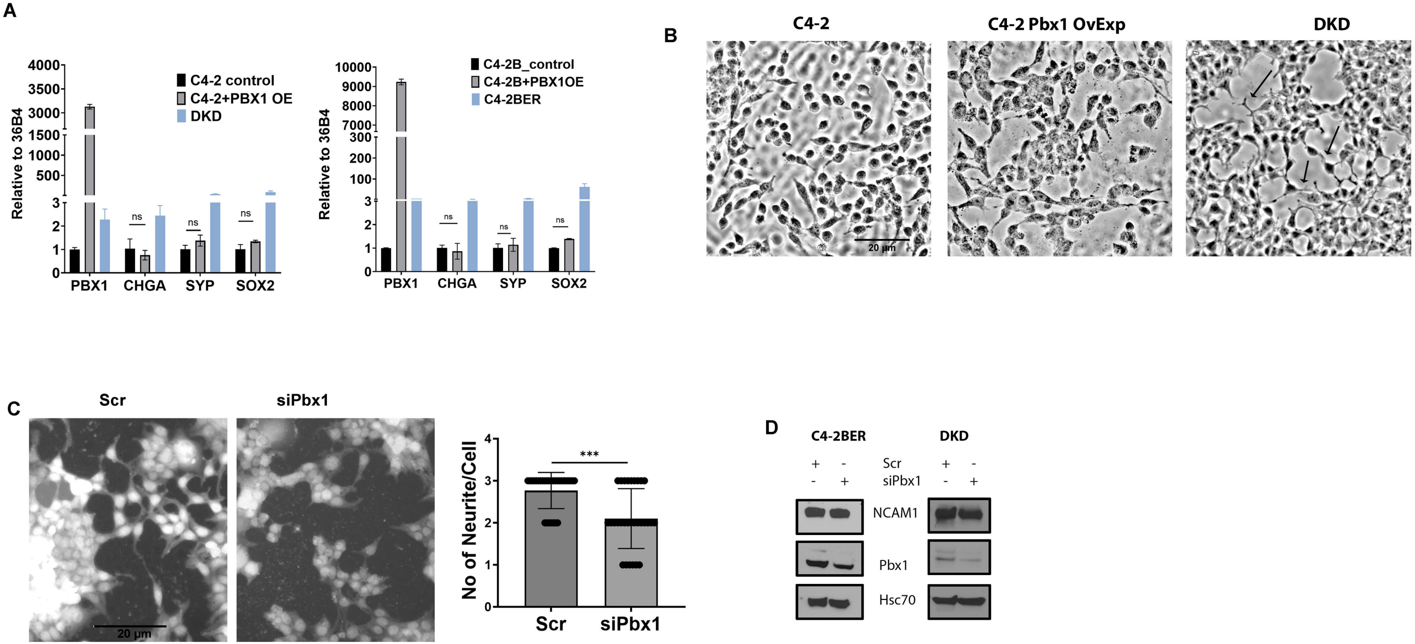

Supplementary Figure 8

Supplementary Table S1: Identification of transcription factor by TomTom Motif search

gene	log2FC
GATA4	9.688737883
SOX5	8.840785039
RUNX3	8.077416012
FLI1	7.422757503
PAX5	7.111410745
FOXA3	6.990671426
IRF8	6.56510523
MEF2C	6.504097934
EGR1	6.465344446
SOX2	6.318717477
FOXC1	5.717093616
ZEB1	4.966592771
ETV5	4.913860326
PRDM6	4.794385418
KLF12	4.694616013
EGR2	4.535970181
ETS1	4.173344498
GATA3	4.161226512
FOXQ1	3.720685531
ZIC1	3.284212781
CDX2	3.177111837
SALL4	3.175333226
MYOD1	3.153441018
SIX2	2.9462795
PBX1	2.168289673
AHR	2.041435187
LEF1	1.768196667
ELF3	1.764638408
ETS2	1.332880981
PRDM1	1.301894707
GFI1	1.276975592

Supplementary Table 2

Probe Ids	logFC	AveExpr	t	P.Value	adj.P.Val	B	C42_AVG	DKD_AVG	deltaBeta
cg00318543	0.321529359	0.690426756	30.1544714	5.52E-06	2.93E-05	4.593530929	0.529662077	0.851191435	0.3215294
cg00464519	0.17262022	0.894009811	22.70469963	1.76E-05	6.98E-05	3.176692794	0.8076997	0.980319921	0.1726202
cg00715929	0.200698345	0.869659693	18.27838955	4.27E-05	0.000141647	2.090434636	0.76931052	0.970008865	0.2006983
cg00953480	0.848509813	0.535635456	100.9866346	3.84E-08	3.03E-06	10.22097891	0.11138055	0.959890363	0.8485098
cg01177580	0.214618079	0.200544504	12.39135795	0.000206363	0.000536173	0.150408372	0.093235465	0.307853544	0.2146181
cg01375009	0.558551234	0.409765223	24.74743585	1.24E-05	5.32E-05	3.607765737	0.130489607	0.68904084	0.5585512
cg02034102	-0.395083297	0.424009992	-21.56714881	2.17E-05	8.23E-05	2.919288646	0.621551641	0.226468344	-0.3950833
cg02126968	0.65101348	0.4368075	69.15284695	1.82E-07	4.02E-06	8.588440801	0.11130076	0.76231424	0.6510135
cg02152475	0.375659205	0.734459219	43.56406545	1.22E-06	1.07E-05	6.405607506	0.546629617	0.922288822	0.3756592
cg02906335	0.219838745	0.162240692	13.21796278	0.000159071	0.000428287	0.471220128	0.05232132	0.272160065	0.2198387
cg03081635	0.162837531	0.861209623	18.3046605	4.25E-05	0.000140963	2.097628207	0.779790858	0.942628389	0.1628375
cg03261800	0.47683898	0.469832265	34.00889124	3.37E-06	2.07E-05	5.190255636	0.231412775	0.708251755	0.476839
cg03273872	0.382564186	0.739005499	32.84134552	3.89E-06	2.28E-05	5.017292219	0.547723406	0.930287592	0.3825642
cg03298495	0.53924173	0.514060731	39.46289854	1.83E-06	1.38E-05	5.922862097	0.244439866	0.783681596	0.5392417
cg03364442	0.323726715	0.780544859	40.73739121	1.61E-06	1.27E-05	6.078474049	0.618681501	0.942408216	0.3237267
cg04503281	0.061695078	0.940160561	6.787884076	0.002218147	0.004476492	-2.769868903	0.909313022	0.9710081	0.0616951
cg04918364	0.527454863	0.393998403	23.55010318	1.52E-05	6.21E-05	3.35968587	0.130270971	0.657725835	0.5274549
cg05516817	0.551796268	0.648141869	29.6095074	5.94E-06	3.09E-05	4.502798883	0.372243735	0.924040003	0.5517963
cg05697028	0.383581545	0.465798762	37.47140552	2.26E-06	1.58E-05	5.668585372	0.27400799	0.657589535	0.3835815
cg05728973	0.082077222	0.928119989	7.016373203	0.001953313	0.003987122	-2.6142024	0.887081378	0.9691586	0.0820772
cg06066240	0.649807656	0.626807036	38.8062383	1.96E-06	1.44E-05	5.840565175	0.301903208	0.951710864	0.6498077
cg06204376	0.669388339	0.516166919	49.04547666	7.49E-07	8.05E-06	6.978522045	0.18147275	0.850861089	0.6693883
cg06272555	0.051960809	0.039492839	4.406551553	0.010913349	0.019350194	-4.705265077	0.013512435	0.065473244	0.0519608
cg06394247	-0.284328656	0.678918648	-18.13378142	4.41E-05	0.000145459	2.050653016	0.821082976	0.53675432	-0.2843287
cg06671087	0.129666165	0.300740956	13.21311145	0.000159307	0.000428822	0.469394161	0.235907874	0.365574039	0.1296662
cg06671450	-0.313180298	0.275668874	-27.87824667	7.61E-06	3.69E-05	4.202662469	0.432259024	0.119078725	-0.3131803
cg06751742	0.406117241	0.318873362	19.02104403	3.63E-05	0.000124072	2.289933166	0.115814742	0.521931983	0.4061172
cg07105962	0.311378044	0.605363125	9.761884963	0.00053586	0.001241817	-1.025255346	0.449674103	0.761052147	0.311378
cg07291719	0.127524158	0.872907375	14.63133001	0.000105489	0.000301142	0.977395681	0.809145296	0.936669454	0.1275242
cg07378893	0.031762023	0.952145808	3.304066362	0.028584693	0.047035049	-5.851039771	0.936264796	0.96802682	0.031762
cg07716113	0.385409421	0.647526963	29.57262381	5.97E-06	3.10E-05	4.496595687	0.454822253	0.840231674	0.3854094
cg07793395	0.513177219	0.696133516	25.99788001	1.01E-05	4.57E-05	3.85413052	0.439544906	0.952722125	0.5131772
cg08111360	0.29806791	0.761869841	26.65006069	9.15E-06	4.23E-05	3.97786168	0.612835886	0.910903796	0.2980679
cg08132344	0.508440726	0.306609369	35.42564343	2.85E-06	1.85E-05	5.391936306	0.052389005	0.560829732	0.5084407
cg08526796	0.133932216	0.208719447	8.235949554	0.001048048	0.002266253	-1.850366856	0.141753339	0.275685555	0.1339322
cg08748127	-0.311043451	0.638285211	-32.10228396	4.27E-06	2.44E-05	4.90444255	0.793806936	0.482763485	-0.3110435
cg09213759	0.512832158	0.364846364	39.2279717	1.87E-06	1.40E-05	5.893589293	0.108430285	0.621262443	0.5128322
cg09737661	0.394712011	0.624104345	36.45799037	2.53E-06	1.71E-05	5.533599892	0.42674834	0.821460351	0.394712
cg09863596	0.148202751	0.590240184	12.90964733	0.00017496	0.000464899	0.353872954	0.516138808	0.664341559	0.1482028
cg10059959	0.215736572	0.817902651	16.40211501	6.64E-05	0.000203814	1.54825141	0.710034365	0.925770936	0.2157366
cg10193836	0.068673832	0.414973764	3.249817453	0.030114249	0.04936318	-5.912235235	0.380636848	0.449310679	0.0686738
cg10769767	0.527552072	0.574419394	53.78193039	5.13E-07	6.54E-06	7.418888115	0.310643358	0.83819543	0.5275521
cg10815316	0.326424436	0.76288669	18.8553644	3.76E-05	0.000127723	2.24610684	0.599674472	0.926098908	0.3264244
cg11360963	0.125872209	0.836936585	6.982533331	0.001989987	0.004055625	-2.636984419	0.774000481	0.899872689	0.1258722
cg11956655	0.667192237	0.512200403	23.90600245	1.43E-05	5.93E-05	3.434739879	0.178604284	0.845796521	0.6671922
cg11998388	0.571479971	0.430021131	54.42219347	4.88E-07	6.37E-06	7.475034592	0.144281145	0.715761116	0.57148
cg12278961	0.58697666	0.649625298	33.10762091	3.76E-06	2.23E-05	5.057300548	0.356136968	0.943113628	0.5869767
cg12348385	0.589035064	0.549177817	44.27907383	1.14E-06	1.03E-05	6.484703891	0.254660285	0.843695349	0.5890351
cg13394473	0.541747003	0.694688899	47.0035619	8.92E-07	8.91E-06	6.773760059	0.423815397	0.9655624	0.541747
cg13482308	0.27792126	0.845679875	34.41207269	3.21E-06	2.00E-05	5.248538608	0.706719244	0.984640505	0.2779213
cg13529513	0.321741537	0.303541371	34.45044962	3.19E-06	2.00E-05	5.254048778	0.142670602	0.46441214	0.3217415
cg13675835	0.145783104	0.13546278	13.70684231	0.000137369	0.000377513	0.65199106	0.062571228	0.208354332	0.1457831
cg13690812	0.407954264	0.332048037	24.14399276	1.37E-05	5.75E-05	3.484298484	0.128070905	0.536025169	0.4079543
cg13701768	0.223174318	0.825728458	17.10932096	5.59E-05	0.00017663	1.759482474	0.714141299	0.937315617	0.2231743
cg13751548	0.525626709	0.586906575	28.96250265	6.51E-06	3.30E-05	4.39280592	0.324093221	0.84971993	0.5256267
cg13774019	0.544570009	0.537593612	41.50789227	1.49E-06	1.21E-05	6.170026403	0.265308608	0.809878616	0.54457
cg13910860	-0.163456201	0.105164084	-21.9892653	2.01E-05	7.73E-05	3.016368537	0.186892184	0.023435983	-0.1634562
cg13959831	0.102154439	0.195193947	13.18012922	0.000160922	0.000432554	0.456962989	0.144116727	0.246271166	0.1021544
cg14094646	0.13811101	0.870097492	18.16706412	4.38E-05	0.000144558	2.059836894	0.801041986	0.939152997	0.138111
cg14099718	0.376420858	0.653473151	38.16563678	2.10E-06	1.51E-05	5.758831762	0.465262722	0.84168358	0.3764209
cg14267095	0.169249444	0.496164833	10.97128435	0.000336356	0.000822096	-0.451651262	0.411540111	0.580789555	0.1692494
cg14283649	0.395463323	0.721972563	46.32006922	9.47E-07	9.23E-06	6.703010451	0.524240901	0.919704225	0.3954633
cg14306956	-0.085331511	0.115518505	-8.109896284	0.001113281	0.002393057	-1.924544306	0.158184261	0.07285275	-0.0853315
cg14317609	-0.329667198	0.249314771	-11.31286423	0.000297495	0.000737891	-0.30036631	0.41414837	0.084481172	-0.3296672
cg14344261	-0.179310426	0.09602182	-16.58136063	6.35E-05	0.000196476	1.602626157	0.185677033	0.006366607	-0.1793104
cg14383078	0.219786821	0.790423074	15.42909007	8.51E-05	0.000251043	1.242498059	0.680529663	0.900316484	0.2197868
cg14442730	0.659135297	0.617216259	53.63255479	5.19E-07	6.58E-06	7.405679425	0.28764861	0.946783907	0.6591353
cg14478307	0.06412678	0.951057967	7.75702799	0.001324385	0.002800416	-2.137746518	0.918994577	0.983121357	0.0641268
cg14498946	0.34113307	0.745435046	21.18131346	2.34E-05	8.72E-05	2.828865025	0.574868511	0.916001581	0.3411331
cg14519598	0.226701796	0.807231474	24.89662112	1.21E-05	5.22E-05	3.637817408	0.693880576	0.920582372	0.2267018
cg14535614	0.580235416	0.623760916	62.83072092	2.71E-07	4.76E-06	8.148577548	0.333643209	0.913878624	0.5802354
cg15063565	0.592694515	0.355144698	47.04650852	8.89E-07	8.89E-06	6.778167542	0.05879744	0.651491955	0.5926945
cg18464558	0.548629947	0.695148219	65.03712698	2.35E-07	4.47E-06	8.307899654	0.420833245	0.969463192	0.5486299
cg18889595	0.425541342	0.235253556	48.0769265	8.13E-07	8.44E-06	6.882600637	0.022482884	0.448024227	0.4255413
cg18928153	0.603716953	0.502253289	12.65805559	0.000189404	0.00049786	0.256102984	0.200394812	0.804111765	0.603717
cg19381899	0.24375291	0.701675563	14.26791274	0.000116796	0.000328468	0.851925777	0.579799108	0.823552018	0.2437529
cg20427438	0.298793929	0.698395885	22.85424607	1.72E-05	6.83E-05	3.209559864	0.548998921	0.84779285	0.2987939
cg20430779	-0.577224077	0.557808014	-71.41210486	1.60E-07	3.84E-06	8.733938467	0.846420053	0.269195976	-0.5772241
cg20700647	0.460906536	0.67392335	44.99962513	1.07E-06	9.90E-06	6.563013148	0.443470082	0.904376617	0.4609065
cg20815683	0.195293765	0.837031619	24.39145432	1.31E-05	5.57E-05	3.535305766	0.739384736	0.934678501	0.1952938
cg23916167	-0.891441256	0.474699654	-119.6986111	1.91E-08	3.03E-06	10.88097647	0.920420282	0.028979026	-0.8914413
cg23917608	-0.210651351	0.292937064	-17.78681225	4.77E-05	0.000155116	1.953903753	0.398262739	0.187611388	-0.2106514
cg24361810	0.332747478	0.597936727	16.65291436	6.24E-05	0.00019359	1.624170755	0.431562988	0.764310466	0.3327475
cg24975610	0.376045379	0.706394535	35.43288501	2.85E-06	1.85E-05	5.392945174	0.518371846	0.894417225	0.3760454
cg25583848	0.164409147	0.101404605	20.00294756	2.96E-05	0.000105083	2.542103733	0.019200031	0.183609178	0.1644091
cg26454672	0.201565285	0.128388792	12.25978103	0.000215422	0.000556587	0.097459384	0.02760615	0.229171435	0.2015653
cg27057201	0.312754911	0.674277293	12.17497337	0.000221522	0.000570276	0.063042918	0.517899838	0.830654748	0.3127549
cg00204390	0.183421656	0.311438945	12.25916753	0.000215465	0.000556678	0.097211231	0.219728117	0.403149773	0.1834217
cg02538199	0.329960345	0.810103182	42.30538027	1.37E-06	1.15E-05	6.262869762	0.645123009	0.975083354	0.3299603
cg03791373	0.207683335	0.772639557	10.27725556	0.000436658	0.001035014	-0.773155021	0.668797889	0.876481224	0.2076833
cg06561106	0.112565175	0.932732877	10.48518674	0.000403132	0.000964519	-0.674754575	0.87645029	0.989015465	0.1125652
cg11551236	0.51561571	0.582122234	41.32179705	1.51E-06	1.22E-05	6.148083369	0.324314379	0.839930089	0.5156157

Probe Ids	CHR	MAPINFO	Strand	Type	gene	feature	cgi	feat.cgi	UCSC_Islands_Name	SNP_ID	SNP_DISTANCE
cg00318543	9	37001111	F	II	PAX5	Body	shore	Body-shore	chr9:37002489-37002957	rs554141824	19
cg00464519	9	37034655	F	II	PAX5	TSS200	island	TSS200-island	chr9:37034135-37038341	rs3758180	14
cg00715929	9	37002528	R	I	PAX5	Body	island	Body-island	chr9:37002489-37002957	rs188164406	31
cg00953480	9	37034933	R	II	PAX5	TSS1500	island	TSS1500-island	chr9:37034135-37038341	rs115331116;rs72735683;rs574359031	24;13;7
cg01177580	9	37011598	F	II	PAX5	Body	opensea	Body-opensea		rs144358127;rs188339597	0;1
cg01375009	9	36966765	F	II	PAX5	Body	opensea	Body-opensea		rs575051854;rs533945481	0;29
cg02034102	9	37026739	R	II	PAX5	Body	island	Body-island	chr9:37026222-37028014	rs548168107;rs145204247;rs527738322;rs547584355	39;30;11;10
cg02126968	9	36934041	R	II	PAX5	Body	opensea	Body-opensea		rs564679280;rs56084540	47;41
cg02152475	9	37029364	R	II	PAX5	Body	shore	Body-shore	chr9:37029932-37030295	rs574072379	22
cg02906335	9	36948026	F	II	PAX5	Body	opensea	Body-opensea		rs552256810	0
cg03081635	9	37002335	F	II	PAX5	Body	shore	Body-shore	chr9:37002489-37002957	rs535575246;rs555405524;rs572487324	5;15;40
cg03261800	9	36985761	F	II	PAX5	Body	shore	Body-shore	chr9:36985986-36986924	rs200266193	9
cg03273872	9	36855131	F	II	PAX5	Body	opensea	Body-opensea		rs138580715	0
cg03298495	9	37006990	R	II	PAX5	Body	opensea	Body-opensea		rs562303869	1
cg03364442	9	36943421	R	II	PAX5	Body	opensea	Body-opensea		rs552167745;rs148029078;rs7853360	51;28;27
cg04503281	9	36856405	F	II	PAX5	Body	opensea	Body-opensea			
cg04918364	9	36875592	F	II	PAX5	Body	opensea	Body-opensea		rs74757610;rs562044301;rs374467713	0;9;18
cg05516817	9	36958157	R	II	PAX5	Body	opensea	Body-opensea		rs111493737;rs573056782;rs544698760;rs564860209;rs113723263;rs10973141	41;34;32;23;8;1
cg05697028	9	36968169	F	II	PAX5	Body	opensea	Body-opensea		rs543026061	4
cg05728973	9	36987377	F	II	PAX5	Body	shore	Body-shore	chr9:36985986-36986924		
cg06066240	9	36896279	F	II	PAX5	Body	opensea	Body-opensea		rs116657740;rs112280673	42;43
cg06204376	9	37034583	F	I	PAX5	TSS200	island	TSS200-island	chr9:37034135-37038341	rs117601198;rs7023441	14;34
cg06272555	9	36862435	F	II	PAX5	Body	opensea	Body-opensea		rs542569566	8
cg06394247	9	36986788	F	II	PAX5	Body	island	Body-island	chr9:36985986-36986924	rs544513193	18
cg06671087	9	37033489	R	II	PAX5	Body	shore	Body-shore	chr9:37034135-37038341	rs555759933;rs572518871	28;13
cg06671450	9	36985987	R	II	PAX5	Body	island	Body-island	chr9:36985986-36986924		
cg06751742	9	36984228	R	II	PAX5	Body	shore	Body-shore	chr9:36985986-36986924		
cg07105962	9	36923549	F	II	PAX5	Body	opensea	Body-opensea			
cg07291719	9	37004134	R	II	PAX5	Body	shore	Body-shore	chr9:37002489-37002957	rs55882407;rs528451445;rs551466310;rs185577105;rs149847851	42;21;4;2;1
cg07378893	9	36850645	F	II	PAX5	Body	opensea	Body-opensea		rs148803446	49
cg07716113	9	36857883	F	II	PAX5	Body	opensea	Body-opensea		rs73648138	0
cg07793395	9	36962759	F	II	PAX5	Body	opensea	Body-opensea		rs559202220	35
cg08111360	9	36851295	R	II	PAX5	Body	opensea	Body-opensea		rs370898534;rs529586907	19;18
cg08132344	9	36942095	R	II	PAX5	Body	opensea	Body-opensea			
cg08526796	9	36901505	R	II	PAX5	Body	opensea	Body-opensea		rs529670704;rs546144173;rs186353467;rs17391944;rs551841762	47;38;37;13;6
cg08748127	9	36913951	R	II	PAX5	Body	opensea	Body-opensea		rs4880034;rs539934723;rs74307355;rs78625005;rs552061247	28;19;17;14;2
cg09213759	9	36929431	F	II	PAX5	Body	opensea	Body-opensea		rs533849711;rs553624604	1;20
cg09737661	9	36855554	R	II	PAX5	Body	opensea	Body-opensea		rs535597888	6
cg09863596	9	36853673	R	II	PAX5	Body	opensea	Body-opensea		rs538886384;rs558735902;rs144322251	31;21;2
cg10059959	9	37035381	R	II	PAX5	TSS1500	island	TSS1500-island	chr9:37034135-37038341	rs116283142	11
cg10193836	9	36912714	F	II	PAX5	Body	opensea	Body-opensea		rs542961934;rs559559193	0;2
cg10769767	9	37024153	F	II	PAX5	Body	shore	Body-shore	chr9:37025492-37025795		
cg10815316	9	37001638	F	II	PAX5	Body	shore	Body-shore	chr9:37002489-37002957	rs200460009;rs577778678	2;16
cg11360963	9	36994340	F	II	PAX5	Body	opensea	Body-opensea		rs529232495;rs543112433	30;33
cg11956655	9	36948337	F	II	PAX5	Body	opensea	Body-opensea			
cg11998388	9	36939317	R	II	PAX5	Body	opensea	Body-opensea		rs10118858;rs111618073;rs542016753	27;2;1
cg12278961	9	37035116	F	II	PAX5	TSS1500	island	TSS1500-island	chr9:37034135-37038341	rs550970775;rs191581100	29;41
cg12348385	9	37034645	F	I	PAX5	TSS200	island	TSS200-island	chr9:37034135-37038341	rs3758180	24
cg13394473	9	37007515	F	II	PAX5	Body	opensea	Body-opensea		rs74670895;rs575800993;rs77593611	13;15;21
cg13482308	9	37002685	R	I	PAX5	Body	island	Body-island	chr9:37002489-37002957	rs570985047	16
cg13529513	9	36880055	R	II	PAX5	Body	shore	Body-shore	chr9:36881950-36882211	rs544843859;rs116776430;rs530069953	51;46;2
cg13675835	9	37030825	R	II	PAX5	Body	shore	Body-shore	chr9:37029932-37030295	rs531167747	50
cg13690812	9	36855636	R	II	PAX5	Body	opensea	Body-opensea		rs138489134;rs76577157;rs141990508	45;22;1
cg13701768	9	36882152	R	II	PAX5	Body	island	Body-island	chr9:36881950-36882211	rs559470837;rs2277143;rs548002046	27;13;9
cg13751548	9	37005935	F	II	PAX5	Body	shelf	Body-shelf	chr9:37002489-37002957	rs545732183	36
cg13774019	9	37029805	R	II	PAX5	Body	shore	Body-shore	chr9:37029932-37030295		
cg13910860	9	36986407	F	II	PAX5	Body	island	Body-island	chr9:36985986-36986924	rs551603130;rs375760913	33;36
cg13959831	9	36938341	R	II	PAX5	Body	opensea	Body-opensea		rs185407930;rs144864667	19;2
cg14094646	9	37030262	F	I	PAX5	Body	island	Body-island	chr9:37029932-37030295	rs532200973;rs61729568	35;37
cg14099718	9	36879449	R	II	PAX5	Body	shelf	Body-shelf	chr9:36881950-36882211	rs558855284;rs186366601;rs537418786	46;42;2
cg14267095	9	37026445	R	I	PAX5	Body	island	Body-island	chr9:37026222-37028014	rs568459292;rs534035394;rs553646524	32;31;7
cg14283649	9	36843876	R	II	PAX5	Body	opensea	Body-opensea		rs4880018;rs539708763;rs556848425	34;7;5
cg14306956	9	37027384	F	I	PAX5	Body	island	Body-island	chr9:37026222-37028014	rs573490387;rs556293485;rs576351659	3;8;37
cg14317609	9	36986006	F	I	PAX5	Body	island	Body-island	chr9:36985986-36986924	rs2277144	7
cg14344261	9	36986380	F	I	PAX5	Body	island	Body-island	chr9:36985986-36986924	rs532065152	22
cg14383078	9	37033750	R	II	PAX5	Body	shore	Body-shore	chr9:37034135-37038341	rs559387891;rs528118501;rs550923066;rs373111915;rs376871896	32;22;7;2;1
cg14442730	9	36928184	R	II	PAX5	Body	opensea	Body-opensea			
cg14478307	9	36937149	F	II	PAX5	Body	opensea	Body-opensea		rs114586141	0
cg14498946	9	36935652	F	II	PAX5	Body	opensea	Body-opensea		rs557700524;rs151121500;rs560720055	0;28;51
cg14519598	9	36906608	F	II	PAX5	Body	opensea	Body-opensea		rs7039962;rs558405640	0;35
cg14535614	9	36861271	R	II	PAX5	Body	opensea	Body-opensea		rs183738501;rs73648147;rs536594757	40;22;21
cg15063565	9	36990742	R	II	PAX5	Body	shelf	Body-shelf	chr9:36985986-36986924	rs191570664;rs542137268	33;21
cg18464558	9	37002911	R	I	PAX5	Body	island	Body-island	chr9:37002489-37002957		
cg18889595	9	37032104	F	II	PAX5	5'UTR	shore	5'UTR-shore	chr9:37029932-37030295	rs577076887;rs145289399;rs562509679;rs3739441;rs548423295	8;33;39;42;43
cg18928153	9	37030203	F	II	PAX5	Body	island	Body-island	chr9:37029932-37030295	rs187218979	14
cg19381899	9	36874057	R	II	PAX5	Body	opensea	Body-opensea			
cg20427438	9	37035179	F	II	PAX5	TSS1500	island	TSS1500-island	chr9:37034135-37038341	rs546727473;rs566583478	11;38
cg20430779	9	36858952	R	II	PAX5	Body	opensea	Body-opensea		rs192760200;rs562449566	23;8
cg20700647	9	37010223	R	II	PAX5	Body	opensea	Body-opensea		rs529318534	2
cg20815683	9	37002510	R	I	PAX5	Body	island	Body-island	chr9:37002489-37002957	rs188164406	13
cg23916167	9	37027267	F	II	PAX5	Body	island	Body-island	chr9:37026222-37028014	rs534592785;rs557617078	16;22
cg23917608	9	36970136	F	II	PAX5	Body	opensea	Body-opensea		rs190650061;rs529870260;rs546772333;rs560468341	0;1;7;19
cg24361810	9	36891986	F	II	PAX5	Body	opensea	Body-opensea		rs189557294	21
cg24975610	9	36949590	R	II	PAX5	Body	opensea	Body-opensea		rs540410970	2
cg25583848	9	36993121	F	II	PAX5	Body	opensea	Body-opensea		rs571617621	29
cg26454672	9	37011472	F	II	PAX5	Body	opensea	Body-opensea		rs944066;rs545398619;rs565426966	7;13;29
cg27057201	9	36874829	R	II	PAX5	Body	opensea	Body-opensea		rs180768125	34
cg00204390	9	37034213	F	I	PAX5	1stExon	island	1stExon-island	chr9:37034135-37038341	rs537048154;rs557472733;rs574012888;rs149582137;rs552724487	1;10;19;42;51
cg02538199	9	37034277	F	II	PAX5	1stExon	island	1stExon-island	chr9:37034135-37038341	rs544928755	36
cg03791373	9	37034233	F	II	PAX5	1stExon	island	1stExon-island	chr9:37034135-37038341	rs149582137;rs552724487;rs147255256	22;31;39
cg06561106	9	37034166	R	II	PAX5	1stExon	island	1stExon-island	chr9:37034135-37038341	rs560811513;rs529500043;rs549263432	37;16;15
cg11551236	9	37034177	F	II	PAX5	1stExon	island	1stExon-island	chr9:37034135-37038341	rs566397475;rs535302981;rs552089001;rs571696527;rs537048154;rs557472733	2;15;25;31;37;46

Probe Ids	Position OF Methylation	CLASSICAL CpG	Sequence (NEED REVERSE COMPLEMENT TO MATCH THE GENOME)	hg38 co-ordinates	hg38 Probe Ids	REVERSE COMPLEMENT		
cg00318543	AC/TG	N		Chr9:37001113-37001115	cg00318543			
cg00464519	GCG	Y	TCTGCGCTG	Chr9:37034657-37034659	cg00464519	CAGCGCAGA		
cg00715929	TCG	Y		Chr9:37002530-37002532	cg00715929			
cg00953480	GCG	N	GCAGCGCCA	Chr9:37034935-37034937	cg00953480	TGGCGCTGC		
cg01177580	ACG	N		Chr9:37011600-37011602	cg01177580			
cg01375009				Chr9:36966767-36966769	cg01375009			
cg02034102				Chr9:37026741-37026743	cg02034102			
cg02126968				Chr9:36934043-36934045	cg02126968			
cg02152475			TTTCCGGCG	Chr9:37029366-37029368	cg02152475	CGCCGGAAA		
cg02906335				Chr9:36948028-36948030	cg02906335			
cg03081635				Chr9:37002337-37002339	cg03081635			
cg03261800				Chr9:36985763-36985765	cg03261800			
cg03273872				Chr9:36855133-36855135	cg03273872			
cg03298495				Chr9:37006992-37006994	cg03298495			
cg03364442				Chr9:36943423-36943425	cg03364442			
cg04503281				Chr9:36856407-36856409	cg04503281			
cg04918364				Chr9:36875594-36875596	cg04918364			
cg05516817				Chr9:36958159-36958161	cg05516817			
cg05697028				Chr9:36968171-36968173	cg05697028			
cg05728973				Chr9:36987379-36987381	cg05728973			
cg06066240				Chr9:36896281-36896283	cg06066240			
cg06204376	GCG	Y	GAAGCGGAG	Chr9:37034585-37034587	cg06204376	CTCCGCTTC		
cg06272555				Chr9:36862437-36862439	cg06272555			
cg06394247				Chr9:36986790-36986792	cg06394247			
cg06671087			TTTCCGAAT	Chr9:37033491-37033493	cg06671087	ATTCGGAAA		
cg06671450				Chr9:36985989-36985991	cg06671450			
cg06751742				Chr9:36984230-36984232	cg06751742			
cg07105962				Chr9:36923551-36923553	cg07105962			
cg07291719				Chr9:37004136-37004138	cg07291719			
cg07378893				Chr9:36850647-36850649	cg07378893			
cg07716113				Chr9:36857885-36857887	cg07716113			
cg07793395				Chr9:36962761-36962763	cg07793395			
cg08111360				Chr9:36851297-36851299	cg08111360			
cg08132344				Chr9:36942097-36942099	cg08132344			
cg08526796				Chr9:36901507-36901509	cg08526796			
cg08748127				Chr9:36913953-36913955	cg08748127			
cg09213759				Chr9:36929433-36929435	cg09213759			
cg09737661				Chr9:36855556-36855558	cg09737661			
cg09863596				Chr9:36853675-36853677	cg09863596			
cg10059959	TCG	N	TGGTCGCAG	Chr9:37035383-37035385	cg10059959	CTGCGACCA		
cg10193836				Chr9:36912716-36912718	cg10193836			
cg10769767				Chr9:37024155-37024157	cg10769767			
cg10815316				Chr9:37001640-37001642	cg10815316			
cg11360963				Chr9:36994342-36994344	cg11360963			
cg11956655				Chr9:36948339-36948341	cg11956655			
cg11998388				Chr9:36939319-36939321	cg11998388			
cg12278961	GCG	N	TTGGCGGGA	Chr9:37035118-37035120	cg12278961	TCCCGCCAA		
cg12348385	GCG	Y	GTCGCGGCC	Chr9:37034647-37034649	cg12348385	GGCCGCGAC		
cg13394473				Chr9:37007517-37007519	cg13394473			
cg13482308				Chr9:37002687-37002689	cg13482308			
cg13529513				Chr9:36880057-36880059	cg13529513			
cg13675835			ACATCGACT	Chr9:37030827-37030829	cg13675835	AGTCGATGT		
cg13690812				Chr9:36855638-36855640	cg13690812			
cg13701768				Chr9:36882154-36882156	cg13701768			
cg13751548				Chr9:37005937-37005939	cg13751548			
cg13774019			GCCTCGAAG	Chr9:37029807-37029809	cg13774019	CTTCGAGGC	phx1 NOT F	chr9:37,029,804-37,029,812
cg13910860				Chr9:36986409-36986411	cg13910860			
cg13959831				Chr9:36938343-36938345	cg13959831			
cg14094646			CGGGCGGCC	Chr9:37030264-37030266	cg14094646	GGCCGCCCG		chr9:37,030,261-37,030,269
cg14099718				Chr9:36879451-36879453	cg14099718			
cg14267095				Chr9:37026447-37026449	cg14267095			
cg14283649				Chr9:36843878-36843880	cg14283649			
cg14306956				Chr9:37027386-37027388	cg14306956	CCCCGGCGA	phx1 NOT F	chr9:37,027,383-37,027,391
cg14317609				Chr9:36986008-36986010	cg14317609			
cg14344261				Chr9:36986382-36986384	cg14344261			
cg14383078			AGGACGCCA	Chr9:37033752-37033754	cg14383078	TGGCGTCCT		
cg14442730				Chr9:36928186-36928188	cg14442730			
cg14478307				Chr9:36937151-36937153	cg14478307			
cg14498946				Chr9:36935654-36935656	cg14498946			
cg14519598				Chr9:36906610-36906612	cg14519598			
cg14535614				Chr9:36861273-36861275	cg14535614			
cg15063565				Chr9:36990744-36990746	cg15063565			
cg18464558				Chr9:37002913-37002915	cg18464558			
cg18889595	GC/TG	N	GGGGC/TGTGG	Chr9:37032106-37032108	cg18889595	CCACGCCCC		
cg18928153			TCGGCGTTC	Chr9:37030205-37030207	cg18928153	GAACGCCGA		chr9:37,030,202-37,030,210
cg19381899				Chr9:36874059-36874061	cg19381899			
cg20427438	ACG		GAGACGTTG	Chr9:37035181-37035183	cg20427438	CAACGTCTC		
cg20430779				Chr9:36858954-36858956	cg20430779			
cg20700647				Chr9:37010225-37010227	cg20700647			
cg20815683				Chr9:37002512-37002514	cg20815683			
cg23916167	CCG	Y		Chr9:37027269-37027271	cg23916167	GCTCCCGGGGACTC	phx1 NOT F	chr9:37,027,263-37,027,276
cg23917608				Chr9:36970138-36970140	cg23917608			
cg24361810				Chr9:36891988-36891990	cg24361810			
cg24975610				Chr9:3694959236949594	cg24975610			
cg25583848				Chr9:36993123-36993125	cg25583848			
cg26454672				Chr9:37011474-37011476	cg26454672			
cg27057201				Chr9:36874831-36874833	cg27057201			
cg00204390				Chr9:37025104-37025106	cg00890208			
cg02538199				Chr9:36840970-36840972	cg03040108			
cg03791373				Chr9:36997798-36997800	cg03096548			
cg06561106				Chr9:36946714-36946716	cg04569537			
cg11551236				Chr9:36867590-36867592	cg04954410			
