# Supplementary material for: Understanding the function of Pax5 in development of docetaxel-resistant neuroendocrine-like prostate cancers
Source: Cell Death Dis. 2024 Aug 25;15(8):617. doi: 10.1038/s41419-024-06916-y (PMC11345443; doi:10.1038/s41419-024-06916-y)
Supplement: Supplementary file 3 — Raw blot [file 41419_2024_6916_MOESM3_ESM.pdf]

Figure 2D

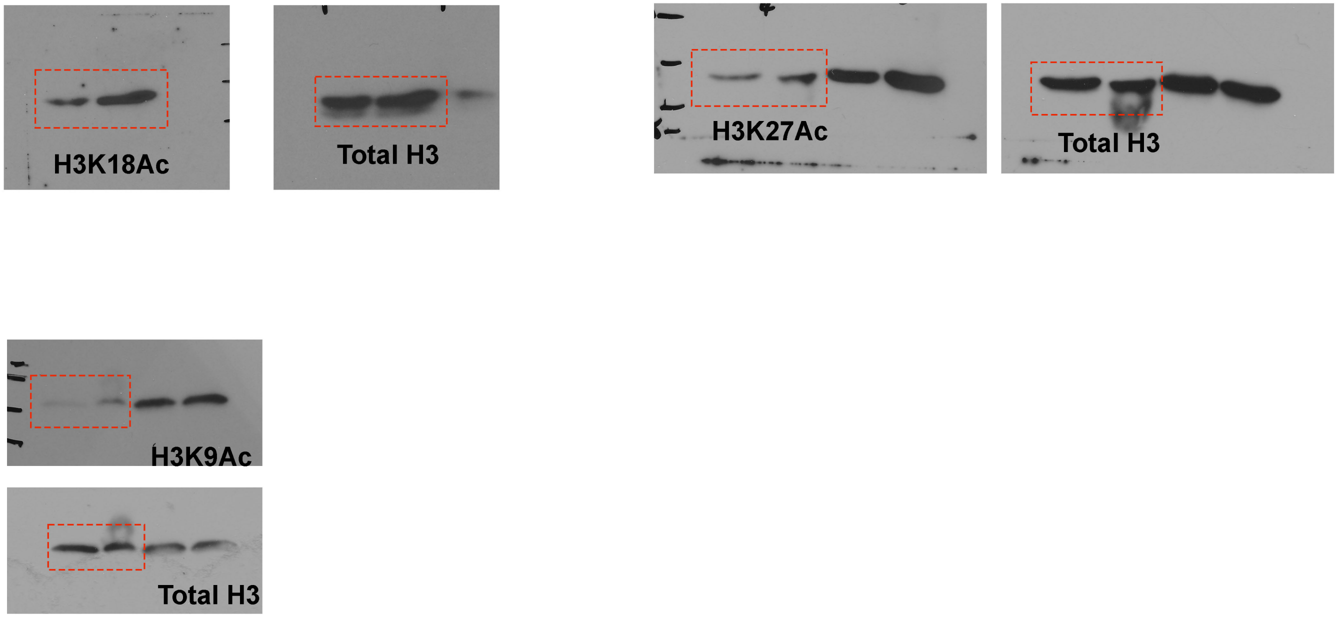

Figure 2

Figure 4A

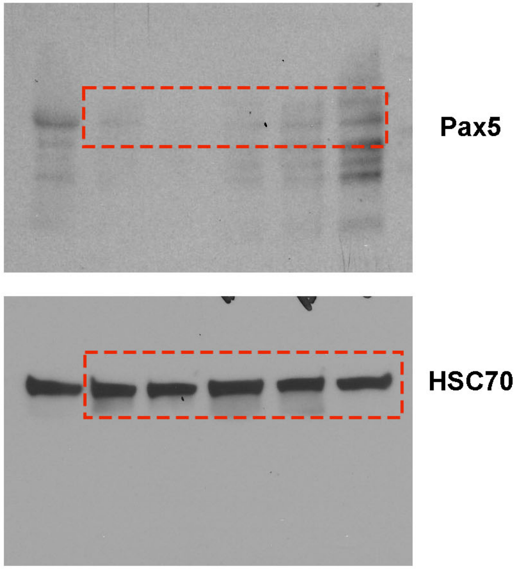

Figure 4

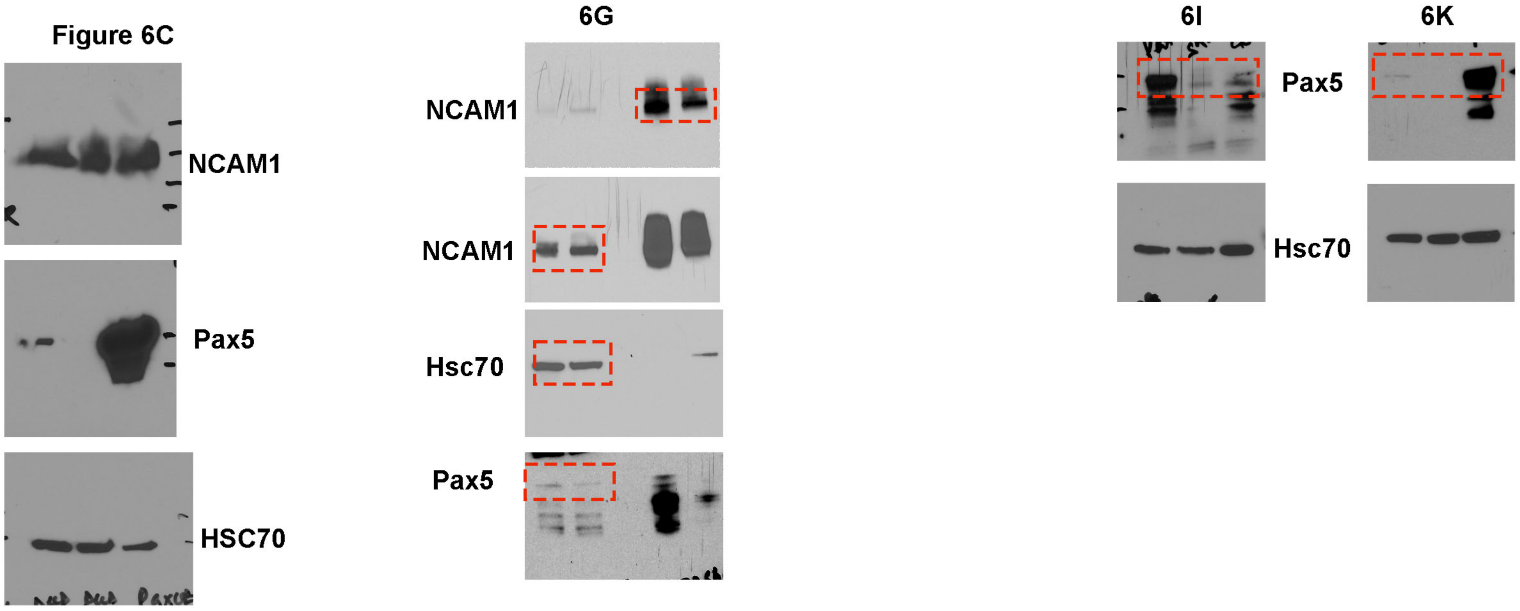
Figure 6C
NCAM1
Pax5
HSC70
6G
NCAM1
NCAM1
Hsc70
Pax5
6I
6K
Pax5
Hsc70

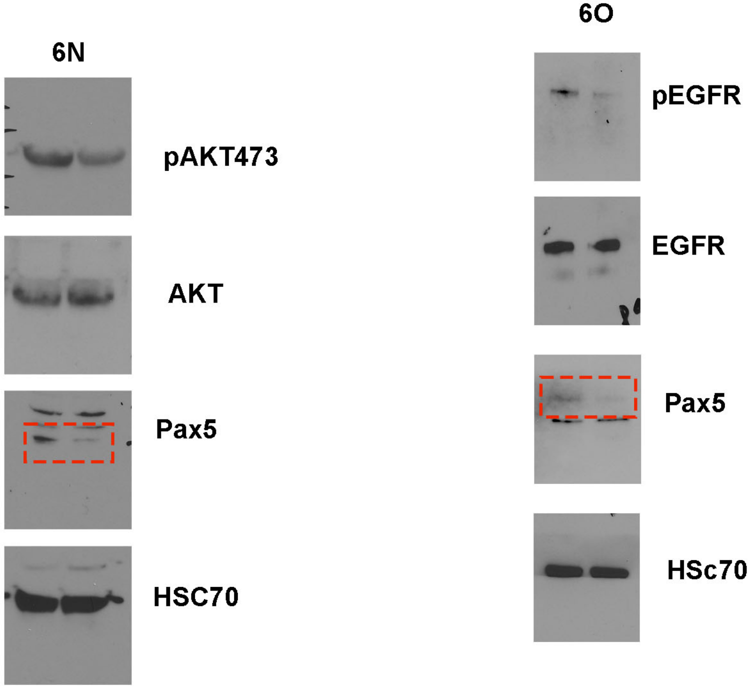
6N
pAKT473
AKT
Pax5
HSC70
6O
pEGFR
EGFR
Pax5
HSc70

Figure 6

7C

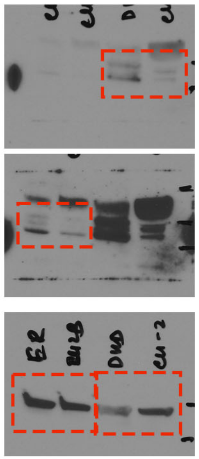

7F

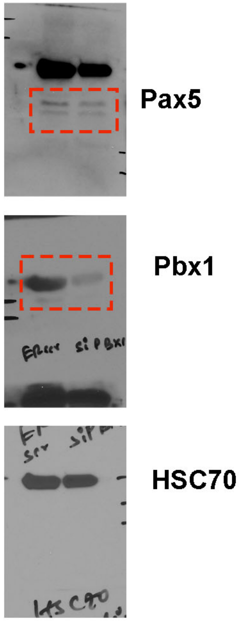

7I

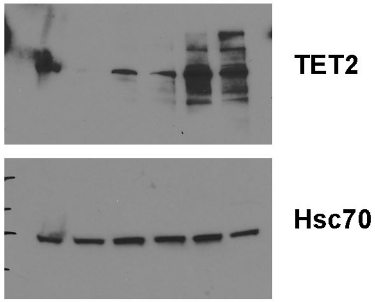

Figure 7

Supplementary Figure 1C

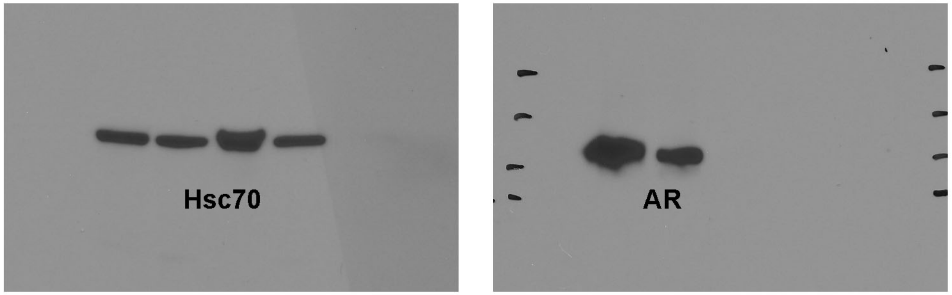

Supplementary Figure 1

Supplementary Fig 2D

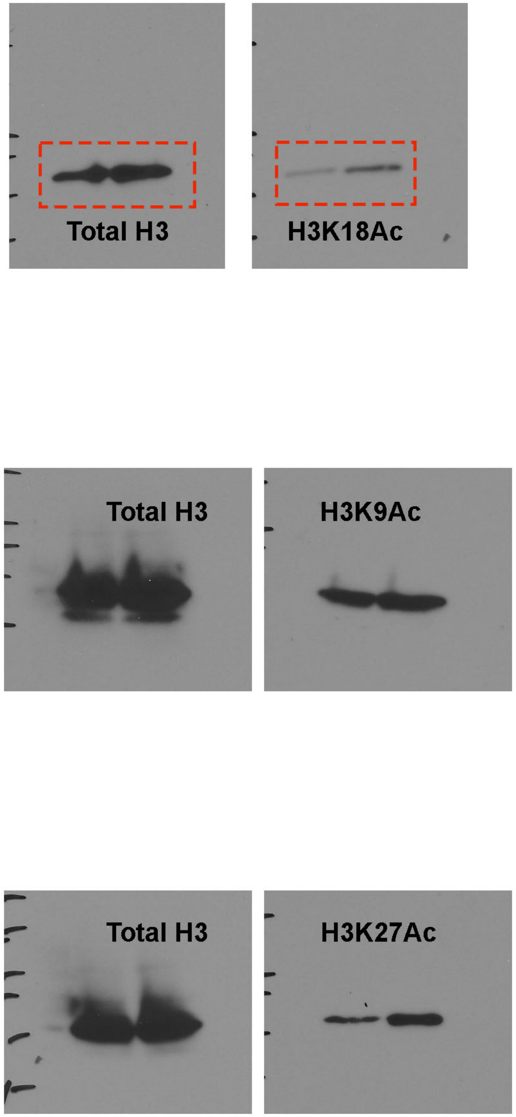

Supplementary Figure 2

S4A

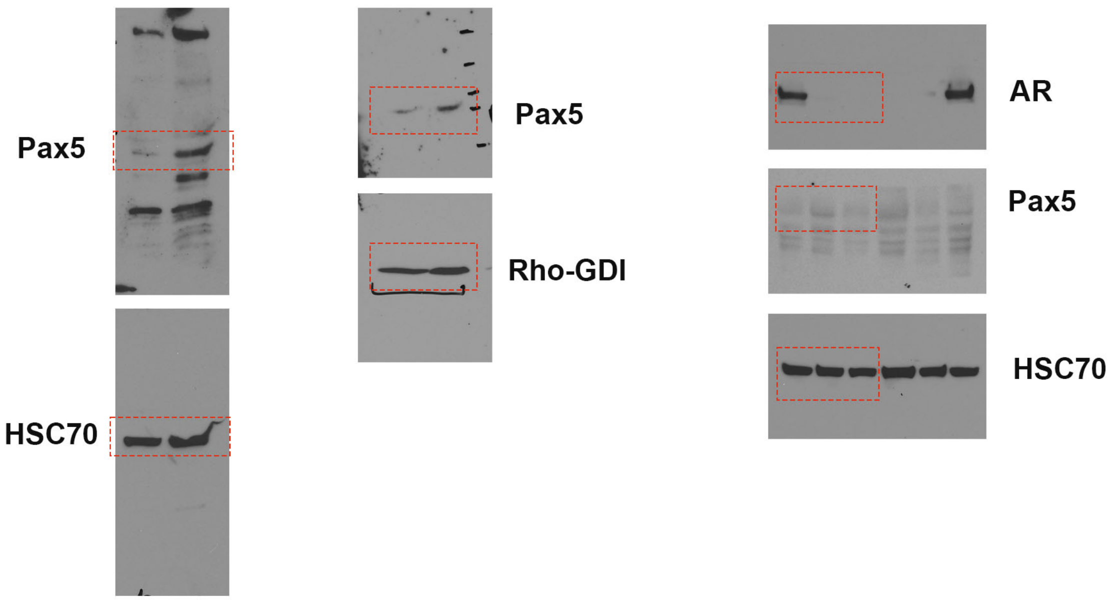

Supplementary fig 4

S5C

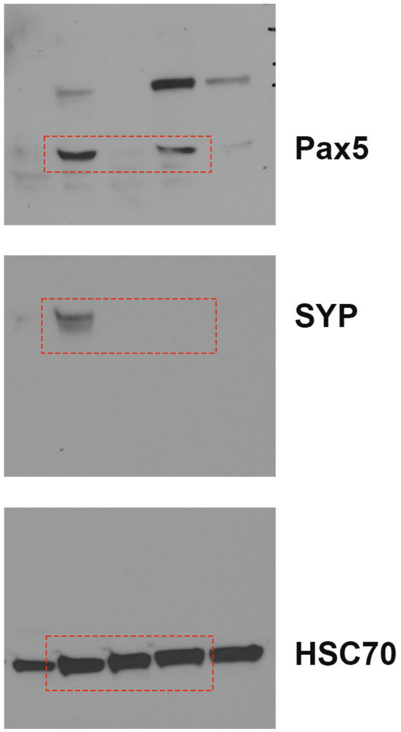

S5D

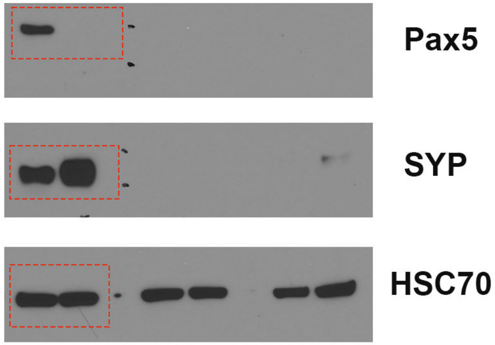

Supplementary fig 5

Suppl Fig 6F

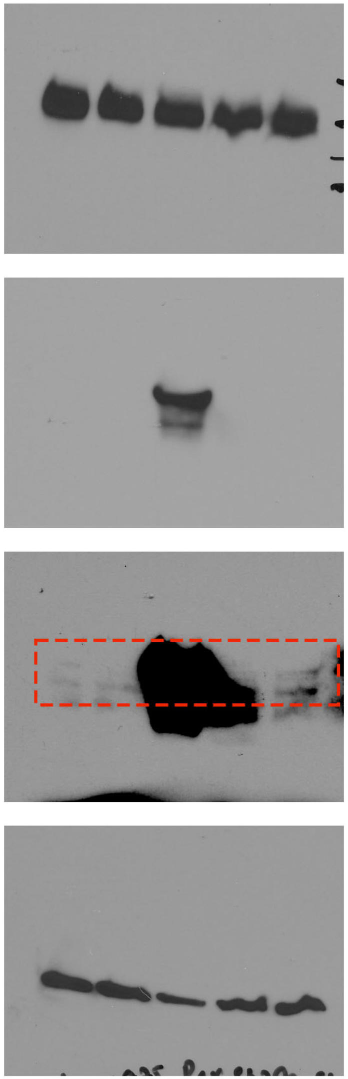

NCAM1

Pax5

Pax5
(darker exposure)

GAPDH

6H

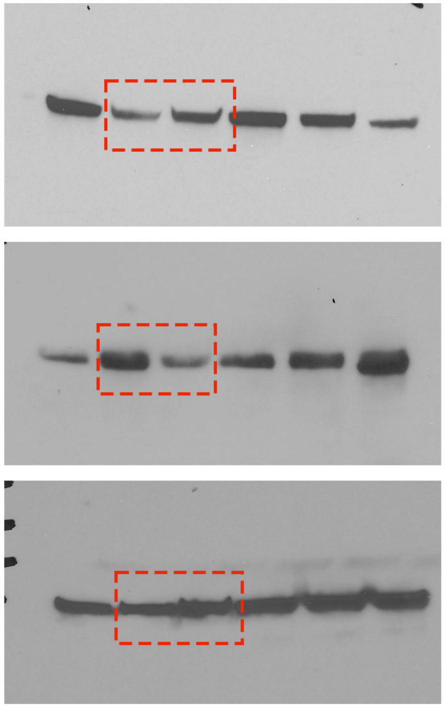

pAKT 473

AKT

HSC70

Supplementary Figure 6

Suppl Fig. 7B

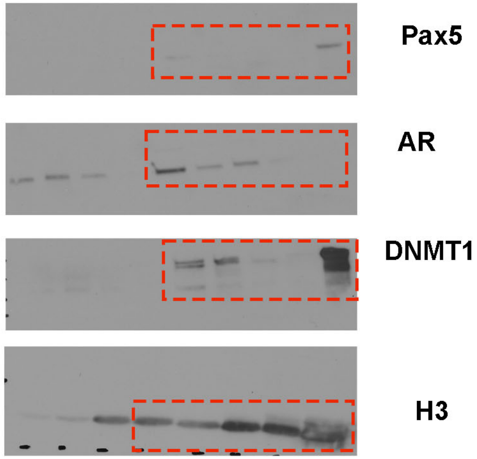

7G

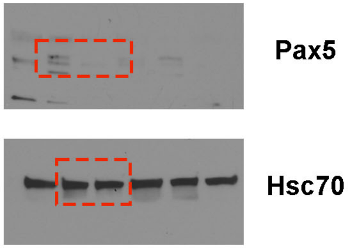

Supplementary figure 7

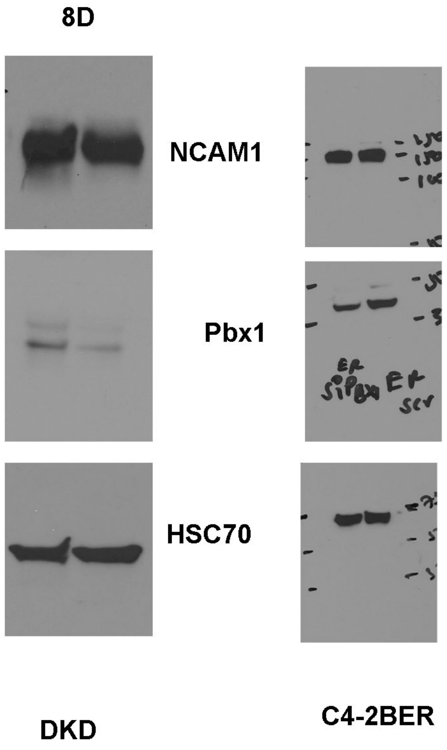

Supplementary Figure 8
